# Calycosin attenuates LPS-induced microglia inflammatory responses and microglia-mediated synaptic impairment via modulation of TLR4/MyD88/NF-κB signaling pathway

**DOI:** 10.3389/fphar.2026.1817366

**Published:** 2026-07-28

**Authors:** Xinran Gao, Ruiyang Wang, Hejuntao Chen, Xu Chen, Xiaojuan Wang

**Affiliations:** 1 The First Affiliated Hospital of Anhui University of Science and Technology, Huainan, China; 2 The First Clinical Medical School, Anhui University of Science and Technology, Huainan, China

**Keywords:** calycosin, microglia, neuroinflammation, neuroprotective, TLR4

## Abstract

**Objective:**

Neuroinflammation plays an important role in the initiation and progression of central nervous system (CNS) disorders. Calycosin (Caly) has been reported to exert anti-inflammatory and neuroprotective effects. However, little is known about its potential role in microglia-mediated neurotoxicity and the underlying mechanism. The aim of the present study is to investigate whether Caly attenuates LPS-induced inflammatory response in BV2 microglial cells and microglia-mediated neuronal injuries in HT-22 cells.

**Methods:**

BV2 cells were stimulated with LPS (1 μg/mL, 24 h) and treated with Caly (5, 10, or 20 μM) or Dex (2 μM). Molecular docking, CETSA, ITDRF_CETSA_ and DARTS assays were used to evaluate the potential target engagement between Caly and TLR4. Cell viability, microglial activation, inflammatory mediator expression, secreted cytokine levels, and TLR4/MyD88/NF-κB signaling pathway were examined. HT-22 cells were cultured with conditioned medium (CM) derived from LPS-stimulated or Caly-treated BV2 cells, cell viability and expression of synaptic plasticity-related proteins were evaluated. Supplementary validation was also performed in HMC3 human microglial cells and SH-SY5Y human neuronal cells.

**Results:**

Stimulation with LPS could induce BV2 microglial activation, accompanied by the increased expression and secretion of pro-inflammatory mediators, and dysregulation of TLR4/MyD88/NF-κB signaling pathway. CM derived from LPS-stimulated BV2 cells decreased HT-22 cell viability and reduced synaptic plasticity-related protein expression levels. Caly treatment attenuated LPS-induced cytotoxicity and inflammatory responses, and reversed imbalanced expression of TLR4/MyD88/NF-κB signaling pathway in LPS-challenged BV2 cells, as indicated by the reduced Iba-1 fluorescence intensity, decreased mRNA expression levels and secretion of IL-1β, IL-6, and TNF-α, and decreased proteins expression of IL-17, TLR4, MyD88, p-NF-κB/NF-κB and p-IKB/IKB. Caly also increased the expression of the anti-inflammatory phenotype-associated markers CD206 and Arg1, while reducing the pro-inflammatory markers CD86 and iNOS. Moreover, CM derived from Caly-treated BV2 cells partially restored HT-22 cell viability and protein expression of β-Tubulin, PSD95, and Synapsin I. Supplementary experiments in HMC3 and SH-SY5Y cells showed similar anti-inflammatory and synaptic protein-preserving effects.

**Conclusion:**

Caly could exert potential effects on suppressing LPS-induced microglia activation and inflammatory responses in immortalized BV2 cells by regulating TLR4/MyD88/NF-κB signaling pathway, and attenuating microglia-mediated neuronal injury and synaptic impairment in immortalized HT-22 cells.

## 1 Introduction

Neuroinflammation generally refers to inflammatory responses within the central nervous system (CNS) (Hoffman et al., 2026), and has emerged as a pivotal pathophysiological hallmark of various disorders, including Alzheimer’s disease (AD), Parkinson’s disease (PD), traumatic brain injury (TBI) and depression (Shi and Yong, 2025). Microglia are innate immune cells of the myeloid lineage that reside in the CNS, which respond rapidly to pathological triggers and serve as the first line of defense in maintaining brain homeostasis (Wright-Jin and Gutmann, 2019). Under physiological conditions, microglia exhibit a highly dynamic homeostatic phenotype and continuously survey the CNS microenvironment. However, once activated, microglia could produce multiple inflammatory cytokines, including interleukin-1β (IL-1β), interleukin-6 (IL-6), and tumor necrosis factor-α (TNF-α), which amplify neuroinflammatory responses and contribute to CNS destabilization and neuronal degeneration (Wright-Jin and Gutmann, 2019). Accumulating evidences have demonstrated that microglia-mediated neuroinflammation plays an important role in the pathogenesis of neurological disorders (Leng and Edison, 2021), which making it a critical therapeutic target for mitigating neuroinflammatory damage.

Recent years, the intricate crosstalk between microglia and neurons have remained a central focus in neuroscience research (Marinelli et al., 2019; Frosch et al., 2025). Sustained microglial activation initiates chronic neuroinflammatory responses that could disturb neuronal health and disrupt communication between neurons and microglia (Jiang et al., 2022; Witcher et al., 2021). It has been reported that persistent immune stimulation could induce microglia to secrete pro-inflammatory cytokines and neurotoxic substances, which may damage synapses, inhibit neurogenesis, and eventually contribute to CNS disorders (Chamera et al., 2020). Reciprocally, neurons could also regulate microglial activation and function by releasing chemokines such as the CX3CL1 (Pereira-Santos et al., 2024). This bidirectional communication is essential for maintaining synaptic integrity and regulating learning and memory processes (Sun et al., 2024), as evidenced by the findings that impaired microglia-neuron crosstalk contributes to cognitive deficits in aging and neurodegenerative diseases (Kang et al., 2025). Thus, preserving the integrity of microglia-neuron crosstalk and preventing microglia-mediated synaptic damage are critical for protecting neuronal function and mitigating the progression of neurological disorders.

Toll-like receptor 4 (TLR4) is an innate immune receptor responsible for recognizing endogenous danger signals, which is extensively expressed in microglia within cognition-associated brain regions, such as the hippocampus and prefrontal cortex (Zusso et al., 2019), and the inflammatory signaling pathway mediated by TLR4 is recognized as a pivotal node in triggering and amplifying neuroinflammation (Heidari et al., 2022; Liu et al., 2020). It has been reported that the binding of endogenous ligands to TLR4 could activate downstream signaling cascades, including the MyD88/nuclear factor kappa B (NF-κB) pathway, thereby promoting microglial polarization towards a pro-inflammatory phenotype (Si et al., 2024) and inducing the robust release of pro-inflammatory cytokines (TNF-α, IL-1β, IL-6), as well as reactive oxygen species (ROS) (Wei et al., 2024). These mediators could not only impair neuronal synaptic plasticity and blood-brain barrier (BBB) integrity, but also disrupt physiological crosstalk between microglia and neurons, leading to synaptic dysfunction, neuronal apoptosis, and ultimately cognitive impairment (Wei et al., 2024; Chen et al., 2022). Moreover, downregulation or knockout of TLR4 has been shown to significantly alleviate neuroinflammation, synaptic damage and cognitive deficits in diseases models of Alzheimer’s disease (AD) and depression (Zhang et al., 2018; Fan et al., 2023). Thus, it is reasonable and necessary to investigate the role of TLR4-associated neuroinflammation in microglia-neuron crosstalk.

As a natural active isoflavonoid, calycosin (Caly) is one of the main components isolated from Astragalus membranaceus (Gong et al., 2021), which exhibits diverse pharmacological activities, including anti-inflammatory, antioxidant and neuroprotective effects (Deng et al., 2021; Gong et al., 2021). Previous studies have confirmed that Caly could alleviate chronic inflammation in diseases such as rheumatoid arthritis and obesity by inhibiting the TLR4/NF-κB pathway and suppressing the release of pro-inflammatory cytokines, including TNF-α and IL-1β (Zhu et al., 2021; Tian et al., 2022). Moreover, in the CNS, Caly has been reported to reduce neuronal damage and improve cognitive function in disease models of cerebral ischemia and AD through antioxidant effects, attenuation of neuroinflammation and inhibition of neuronal apoptosis (Xu S. et al., 2023; An et al., 2022). However, although recent studies have indicated that Caly may exert anti-neuroinflammatory effects by targeting the TLR4/NF-κB signaling pathway (Yang et al., 2019; Li et al., 2023), the regulatory effects of Caly on LPS-induced microglial inflammation and microglia-neuron crosstalk remain unclear.

Given the critical role of microglial activation and synaptic damage in neuroinflammatory disorders, exploring the potential neuroprotective mechanisms of Caly in regulating microglia-neuron crosstalk is of considerable research value. Therefore, the present study aimed to investigate the effects of Caly on LPS-induced microglial inflammatory responses and microglia-mediated synaptic impairment in neuronal cells. BV2 cells were stimulated with LPS and treated with different concentrations of Caly, and a BV2/HT-22 conditioned medium-based indirect co-culture system was established. Cell viability and inflammatory mediators were detected. The expression of proteins involved in the TLR4/MyD88/NF-κB signaling pathway and key molecular related to synaptic plasticity were measured. Our findings might provide experimental evidence for the potential pharmacological relevance of Caly in neuroinflammation-related CNS disorders.

## 2 Materials and methods

### 2.1 Drugs and reagents

Lipopolysaccharide (LPS) was purchased from Sigma Chemical Co. (United States). Calycosin (Caly) and Dexamethasone (Dex) were purchased from Med Chem Express (United States). Phosphate buffer saline (PBS) and 0.25% trypsin-EDTA were purchased from Wuhan Saiweier Biotechnology Co. (China). Both Caly and Dex were dissolved in dimethyl sulfoxide (DMSO, Beyotime Biotechnology, Shanghai, China) and diluted with DMEM before use.

### 2.2 Cell culture

The mouse microglial cell line BV2 and mouse hippocampal neuronal cell line HT-22 were obtained from the China Center for Type Culture Collection (Wuhan, China). Cells were maintained in BV2 cell-specific complete medium (DMEM, SNLM-155, Sunncell) and HT-22 cell-specific complete medium (DMEM, SNLM-202, Sunncell) respectively, and incubated at 37 °C in a 5% carbon dioxide incubator.

### 2.3 Microglial cell activation and treatment

LPS was used to establish an overactivated microglia cellular model. Briefly, exponentially growing BV2 cells (5 × 10^5^ cells/well) were seeded in 6-well plates and cultured until they reached approximately 60% confluence. The cells were then treated with LPS at a concentration of 1 μg/mL for 24 h. Morphological changes in the cells were observed under a phasecontrast microscope. To evaluate the protective effects of Caly against microglia-mediated inflammatory response, BV2 cells were pretreated with Caly (5, 10, or 20 μM) or Dex (2 μM) for 12 h, followed by exposure to LPS (1 μg/mL) for an additional 24 h.

### 2.4 Conditioned medium collection and treatment of HT-22 cells

To prepare BV2 conditioned medium (CM), BV2 cells were treated with Caly (5, 10, 20 μM) and stimulated with LPS (1 μg/mL). The culture supernatants were then collected and filtered through a 0.22 µm filter to remove cells and cell debris. HT-22 cells were seeded in 6-well plates at a density of 5 × 10^5^ cells/well and incubated for 12 h. Subsequently, 50% of the culture medium in each well was removed and replaced with an equal volume of BV2-CM. After incubation for 24 h, HT-22 cells were assessed for cell viability and harvested for protein expression analysis and immunofluorescence staining.

### 2.5 Cell viability detection

Cell viability was evaluated using the methyl thiazolyl diphenyl-tetrazolium bromide (MTT) assay. Briefly, BV2 and HT-22 cells were seeded in 96-well plates at a density of 1 × 10^4^ cells/well and cultured for 12 h to allow attachment. After exposure to LPS, Caly, or corresponding CM for the indicated time, 20 μL of MTT solution (5 mg/mL) was added to each well, followed by incubation for another 4 h. Subsequently, the culture medium was removed, and 150 μL of dimethyl sulfoxide (DMSO) was added to dissolve the formazan crystals. The absorbance was determined at 490 nm using a microplate reader, and cell viability was calculated as a percentage relative to the untreated control group. All experiments were performed in triplicate to ensure reproducibility.

### 2.6 Molecular docking procedure

The three-dimensional structure of human TLR4 was retrieved from the Protein Data Bank (PDB ID: 2Z65), which corresponds to the TLR4-MD-2 complex co-crystallized with the antagonist eritoran. To specifically target the TLR4 - MD-2 interaction interface, the active site was defined based on previously reported key residues (Asp-209, Ser-183) and the MD-2 Gly-97 - Leu-108 loop region (Švajger et al., 2013). The receptor structure was prepared by removing water molecules, heteroatoms, and the bound ligand, followed by addition of polar hydrogens and assignment of Gasteiger charges using AutoDockTools (version 1.5.7).

The three-dimensional structure of Caly (SUB1) was constructed using ChemDraw and energy-minimized with the MMFF94 force field in OpenBabel. The ligand was treated as flexible, while the receptor was kept rigid. Molecular docking was performed using AutoDock Vina (version 1.1.2). A cubic search box with dimensions of 18.0 Å × 18.0 Å × 18.0 Å was centered at coordinates x, y, z = (23.75, 15.11, 30.38), which fully covered the previously reported TLR4 binding cleft involving Asp-209 and Ser-211. The following parameters were applied: num_modes = 10, energy_range = 8, exhaustiveness = 10, and cpu = 16. The binding affinity was calculated using the Vina scoring function, and the best-scoring pose was selected based on the lowest binding energy (kcal/mol). Visualization and analysis of intermolecular interactions (hydrogen bonds, hydrophobic contacts, and electrostatic interactions) were performed using PyMOL (version 2.5) and Discovery Studio Visualizer.

### 2.7 Cellular thermal shift assay (CETSA) and isothermal dose-response fingerprint (ITDRF_CETSA_)

For CETSA experiments, exponentially growing BV2 cells were seeded at a density of 5 × 10^5^ cells/well and treated with either Caly (10 μM) or an equivalent volume of DMSO for 4 h. Cells were harvested, pelleted, and resuspended in PBS containing 1 mmol/L PMSF. The cell suspensions were aliquoted and heated in a thermal cycler at temperatures ranging from 37 °C to 72 °C for 3 min, followed by cooling to room temperature. After three freeze-thaw cycles in liquid nitrogen, the soluble protein fractions were collected by centrifugation at 12,000 rcf for 20 min at 4 °C. For ITDRF_CETSA_ experiments, the procedures were similar to those used for CETSA, except that cells were treated with different concentrations of Caly (1–80 μM) and heated at 65 °C. Equal volumes of soluble protein fractions were loaded onto a 10% SDS-PAGE gel, followed by Western blotting. The band intensity of TLR4 was quantified using ImageJ software (National Institutes of Health, United States) and normalized to Actin. CETSA and ITDRF_CETSA_ curve analyses was performed using GraphPad Prism 6.0 (San Diego, CA, United States).

### 2.8 Drug affinity responsive target stability (DARTS)

Exponentially growing BV2 cells were seeded at a density of 5 × 10^5^ and cultured overnight. Then the cells were performed on ice and lysed with RIPA buffer (Beyotime Biotechnology, Shanghai, China) for 15 min. The protein samples were incubated with Caly (5, 10, or 20 μM) overnight, followed by digestion with pronase E (TargetMol, Shanghai, China) at a 200:1 protein-to-protease ratio. TLR4 protein expression was then analyzed using Western blotting, and DRATS curve analysis was performed using GraphPad Prism 6.0 (San Diego, CA, United States).

### 2.9 Immunofluorescence staining

For immunofluorescence staining, BV2 and HT-22 cells were seeded on glass coverslips and cultured to 60%–70% confluence. After treatment, the cells were fixed with 4% paraformaldehyde for 30 min, permeabilized with 0.3% Triton X-100 (Beyotime Biotechnology, Shanghai, China) for 10 min, and blocked with 10% bovine serum albumin (BSA) for 30 min at room temperature. Then cells were then incubated with primary antibodies (anti-TLR4, anti-MyD88, anti-β-Tubulin or anti-Synapsin I, 1:100) at 4 °C overnight, followed by incubation with the appropriate secondary antibodies (goat anti-rabbit Alexa Fluor 488 or goat anti-mouse Alexa Fluor 647, 1:200) at room temperature for 2 h. 4′,6-diamidino-2-phenylindole (DAPI; Epizyme Biotech, Shanghai, China) was used to counterstain the nucleus. The Coverslips were mounted and observed under the fluorescence microscope (Leica, Germany). The fluorescence images were captured and analyzed to evaluate the expression and localization of the target proteins.

### 2.10 Enzyme-linked immunosorbent assay (ELISA)

The concentrations of secreted IL-1β, IL-6, and TNF-α in the culture supernatant of BV2 cells were quantified using ELISA. Briefly, BV2 cells were seeded in 6-well plates at a density of 5 × 10^5^ cells/well and treated with LPS at 1 μg/mL. After treatment with Caly (5, 10, or 20 μM) or Dex (2 μM), the culture supernatants were collected and centrifuged at 3,000 rpm for 15 min at 4 °C to remove cells and debris. The levels of mouse IL-1β, IL-6, and TNF-α were measured using commercially available ELISA kits according to the manufacturer’s instructions (invitrogen, Thermo Fisher Scientific, Waltham, United States). Standards and samples were added to pre-coated microplates in duplicate or triplicate. After incubation and washing, biotinylated detection antibodies and horseradish peroxidase-conjugated streptavidin were sequentially added, followed by substrate solution. The reaction was stopped using stop solution and absorbance was measured at 450 nm using a microplate reader. Cytokine concentrations were calculated from standard curves generated using recombinant cytokine standards supplied with the kits. The concentrations of IL-1β, IL-6, and TNF-α were expressed as pg/mL.

### 2.11 Quantitative real-time PCR

Total RNA was extracted from BV2 cells using TRIzol reagent. Equal quantities of total RNA were used for qPCR analysis. The mRNA expression levels were measured using a one step SYBR Green PCR kit (AG11732, Accurate Biotechnology Co., Ltd, ChangSha, China) on a LightCycler® 480 system. PCR amplification was performed for 40 cycles. The relative expression levels of target genes were calculated using the 2^−ΔΔCT^ method and normalized to β-actin. The primer sequences used in the present study were provided in Table 1.

**TABLE 1:** The primer sequences.

Source	Gene	Forward primer	Reverse primer
Mouse	IL-1β	GCTTCAGGCAGGCAGTAT	ACA​AAC​CGC​TTT​TCC​ATC​T
	IL-6	GAG​GAT​ACC​ACT​CCC​AAC​AGA​CC	AAG​TGC​ATC​ATC​GTT​GTT​CAT​ACA
	TNF-α	CAT​CTT​CTC​AAA​ATT​CGA​GTG​ACA​A	TGG​GAG​TAG​ACA​AGG​TAC​AAC​CC
	CD206	TGT​ACG​CAG​TGG​TTG​GCA​GTG	GCT​CTG​ATG​ATG​GAC​TTC​CTG​GTA​G
	CD86	CAG​CAC​GGA​CTT​GAA​CAA​CC	CTC​CAC​GGA​AAC​AGC​ATC​TGA
	Arg1	CAG​TCC​CTG​GCT​TAT​GGT​TAC​CC	GAA​GAC​AGC​AGA​GGA​GGT​GAA​GAG
	β-actin	AGT​GTG​ACG​TTG​ACA​TCC​GT	TGC​TAG​GAG​CCA​GAG​CAG​TA

### 2.12 Western blotting

Equal amounts of protein extracted from BV2 and HT-22 cells were used for western blotting analysis. Briefly, cells were lysed with RIPA buffer (Beyotime Biotechnology, Shanghai, China) containing protease and phosphatase inhibitors. Protein concentrations were determined using a BCA kit (Beyotime Biotechnology, Shanghai, China). Equal amounts of proteins (30 μg) were separated by 10% SDS-PAGE gels (Epizyme Biotech, Shanghai, China) and transferred onto polyvinylidene difluoride (PVDF) membranes. The membranes were blocked with 5% milk for 1 h at room temperature and then incubated with primary antibodies at 4 °C overnight. The primary antibodies were as follows: anti-TLR4 and anti-MyD88 (1:1,000; Affinity, Jiangsu, China); anti-iNOS, anti-IL-17, and anti-Iba-1 (1:1,000; ZEN BIO, Chengdu, China); anti-PSD95, anti-Synapsin I, and anti-β-Tubulin (1:1,000; Cell Signaling Technology, United States); anti-p-NF-kB, anti-NF-kB, anti-p-IKB, and anti-IKB (1:1,000; Abcam Technology, United States) and anti-Actin (1:1,000; Zhongshan Biotechnology, Inc., Xian, China). After washing with TBST (Epizyme Biotech, Shanghai, China), the membranes were incubated with the corresponding HRP-conjugated secondary antibodies (1:5,000, Zhongshan Biotechnology, Xian, China) for 1 h at room temperature. Protein bands were visualized using an enhanced chemiluminescence system. Band intensities were quantified using ImageJ software (Wayne Rasband, National Institutes of Health, United States), and relative protein expression levels were normalized to Actin.

### 2.13 Statistical analysis

All experimental data were analyzed and graphed using SPSS Statistics 20.0 (Chicago, IL, United States) and GraphPad Prism 8.0 (San Diego, CA, United States). Differences between groups were evaluated by Student’s t test or one-way analyses of variance (ANOVA) followed by LSD *post hoc* test. The Wilcoxon rank-sum test was conducted to account for potential deviations from normality and small sample size. Data are expressed as mean ± SEM, and *P* < 0.05 was considered statistically significant.

## 3 Results

### 3.1 Inflammatory response in activated BV2 cells induced by LPS

**FIGURE 1:**
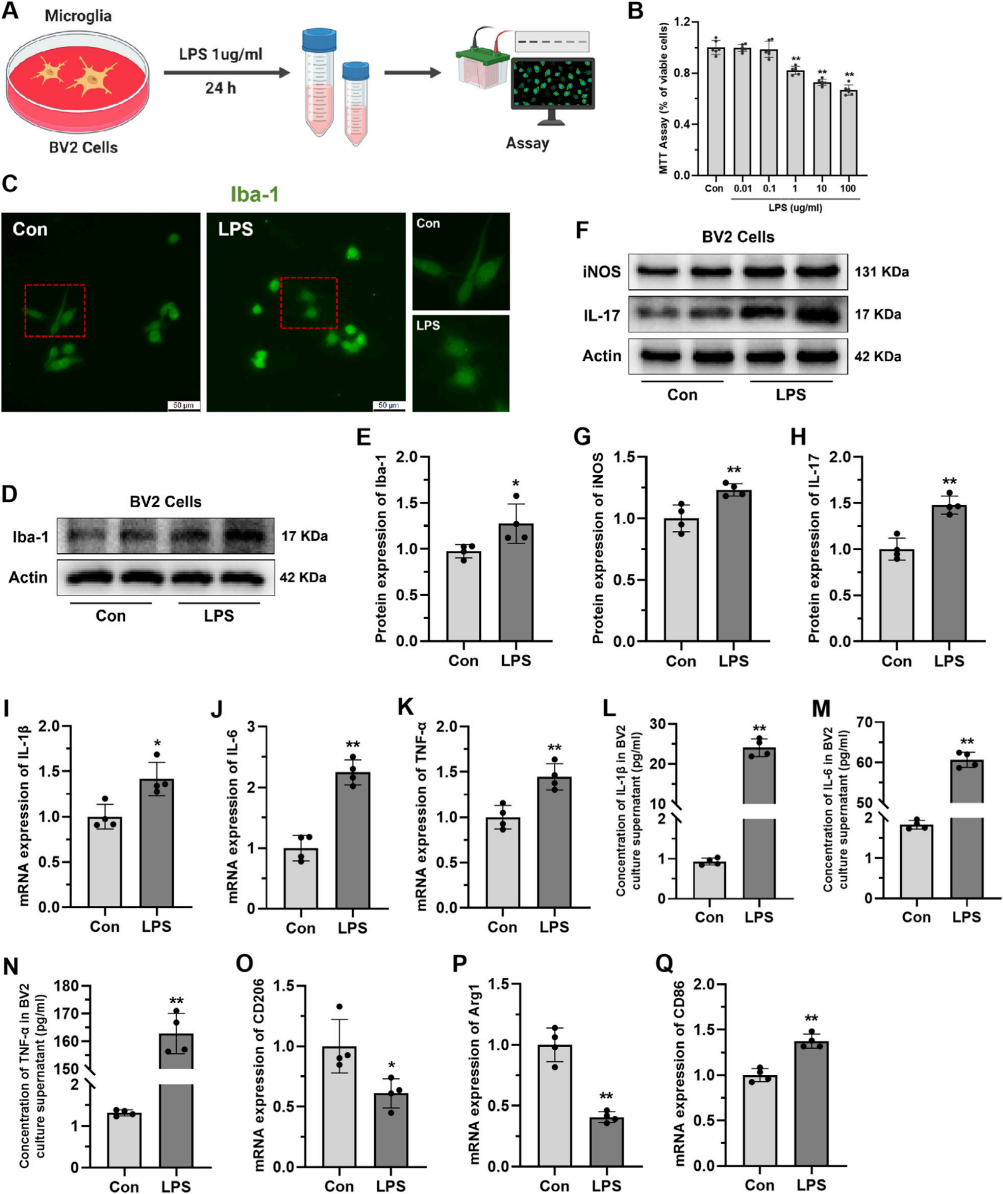
LPS stimulation activates BV2 cells and induces increases inflammatory response. The data are presented as the mean ± SEM, and all the experiments were repeated with two separate passages of cells at least 4 times (n = 4). ^*^ *P* < 0.05 and ^**^ *P* < 0.01 compared with the Con group. **(A)** Outline of the experimental design for BV2 cells. **(B)** Dose effect of LPS (0–100 μg/mL) on the proliferation of BV2 cells. **(C)** Images of immunofluorescence staining for Iba-1 (green) in BV2 cells after LPS stimulation. **(D,E)** Protein expression of Iba-1 in BV2 cells after LPS stimulation. **(F–H)** Protein expression of iNOS and IL-17 in BV2 cells after LPS stimulation. **(I–K)** mRNA expression levels of IL-1β, IL-6 and TNF-α in BV2 cells after LPS stimulation. **(L–N)** Concentrations of IL-1β, IL-6 and TNF-α in the culture supernatant of BV2 cells after LPS stimulation. **(O–Q)** mRNA expression levels of CD206, CD86 and Arg1 in BV2 cells after LPS stimulation.

To establish an *in vitro* model of neuroinflammation, BV2 microglial cells were stimulated with LPS (1 μg/mL) (Figure 1A). Results of MTT assay showed that as compared with the Con group, the cell viability of LPS-challenged BV2 cells were reduced in a dose-dependent manner. Based on these results, 1 μg/mL LPS was selected as the optimal concentration for subsequent stimulation (*P* < 0.01) (Figure 1B). Immunofluorescence staining revealed a marked increase in Iba-1 fluorescence intensity in LPS-induced BV2 cells, accompanied by enlarged cell bodies and short, stout processes, indicating morphological activation (Figure 1C). In addition, the protein expression level of Iba-1 was increased in LPS-challenged BV2 cells (*P* < 0.05), further confirming microglial activation (Figures 1D,E). Additionally, LPS stimulation markedly increased the protein levels of pro-inflammatory mediator iNOS (*P* < 0.01) and inflammation-associated mediator IL-17 (*P* < 0.01) in BV2 cells (Figures 1F–H).

Consistently, the mRNA expression levels of IL-1β (*P* < 0.05), IL-6 (*P* < 0.01) and TNF-α (*P* < 0.01) were significantly upregulated in LPS-challenged BV2 cells relative to the Con ones, as shown in Figures 1I–K. To further confirm the inflammatory response at the secreted protein level, ELISA was performed to quantify cytokine concentrations in BV2 culture supernatant. The results showed that the concentrations of IL-1β (*P* < 0.01), IL-6 (*P* < 0.01), and TNF-α (*P* < 0.01) in the culture supernatant of LPS-challenged BV2 cells were significantly increased as compared with the Con group (Figures 1L–N), which confirming the secretion of pro-inflammatory cytokines into the CM and supporting the successful induction of inflammatory activation. Similar inflammatory responses were also observed in LPS-induced HMC3 cells, as evidenced by the elevated expression of pro-inflammatory cytokines (*P* < 0.01), as shown in Supplementary Figure S1.

Furthermore, LPS stimulation significantly downregulated the mRNA expression levels of CD206 (*P* < 0.05) and Arg1 (*P* < 0.01), which were used as markers of anti-inflammatory microglial phenotypes, in LPS-challenged BV2 cells as compared with the Con group, as shown in Figures 1O,P. In contrast, the mRNA expression level of CD86, a marker associated with pro-inflammatory microglial activation, was markedly increased following LPS challenge (*P* < 0.01) (Figure 1Q). Collectively, these findings indicated that the pro-inflammatory microglial cell model was successfully established.

### 3.2 Dysregulation of TLR4/MyD88/NF-κB signaling pathway in activated BV2 cells induced by LPS

**FIGURE 2:**
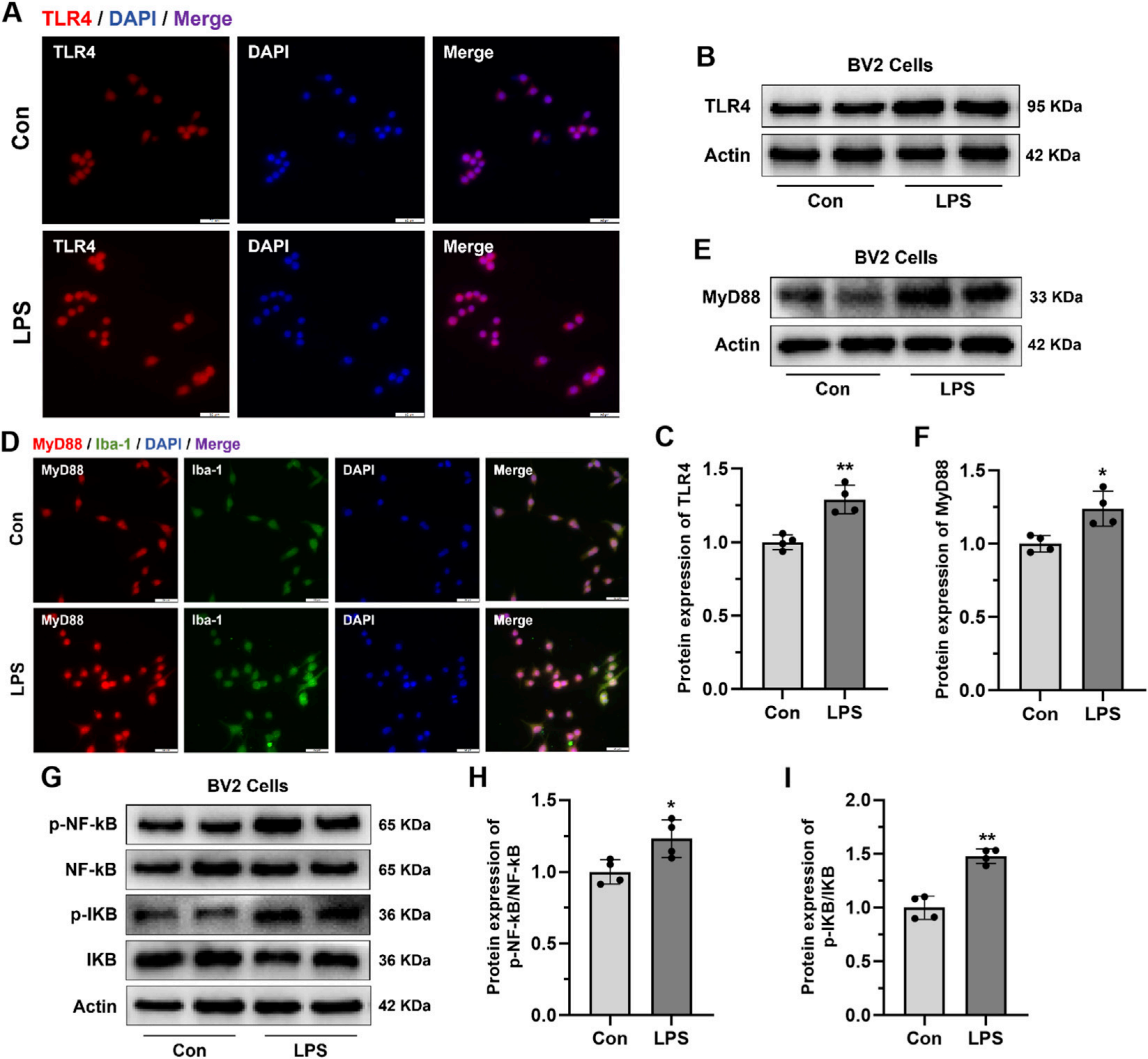
Imbalanced expression of TLR4/MyD88/NF-κB signaling pathway in activated BV2 cells stimulated with LPS. The data are presented as the mean ± SEM, and all the experiments were repeated with two separate passages of cells at least 4 times (n=4). ^*^ *P* < 0.05 and ^**^ *P* < 0.01 compared with the Con group. **(A)** Images of immunofluorescence staining for TLR4 (red) in BV2 cells after LPS stimulation. **(B,C)** Protein expression of TLR4 in BV2 cells after LPS stimulation. **(D)** Images of immunofluorescence double staining for MyD88 (red) and Iba-1 (green) in BV2 cells after LPS stimulation. **(E,F)** Protein expression of MyD88 in BV2 cells after LPS stimulation. **(G–I)** Protein expression of p-NF-kB/NF-kB and p-IKB/IKB in BV2 cells after LPS stimulation.

As shown in Figures 2A–C, LPS stimulation markedly increased TLR4 fluorescence intensity (Figure 2A) and upregulated the protein expression level of TLR4 in BV2 cells compared with the Con group (*P* < 0.01) (Figures 2B,C). Next, double immunofluorescence staining for MyD88 and Iba-1 was performed to further evaluate MyD88 expression in activated microglia. The result showed that the positive fluorescence intensity of MyD88/Iba-1 was significantly increased in LPS-induced BV2 cells compared with Con cells (Figure 2D). Consistently, Western blotting analysis showed that the protein expression level of MyD88 was also increased in LPS-induced cells than the Con cells (*P* < 0.05) (Figures 2E,F). Moreover, increased protein expression levels of TLR4 (*P* < 0.01) and MyD88 (*P* < 0.01) were also observed in LPS-induced HMC3 cells, as shown in Supplementary Figure S2.

Figures 2G–I shows the protein expression levels of phosphorylated NF-kB and IKB in BV2 cells. Compared with the Con group, the relative protein expression levels of p-NF-κB/NF-κB (*P* < 0.05) (Figure 2H) and p-IKB/IKB (*P* < 0.01) (Figure 2I) were both increased in LPS-challenged cells. Taken together, these results suggested that LPS stimulation could induce activation of the TLR4/MyD88/NF-κB signaling pathway in BV2 cells.

### 3.3 CM derived from LPS-challenged microglial cells induced neuronal damage and synaptic plasticity impairment in HT-22 cells

**FIGURE 3:**
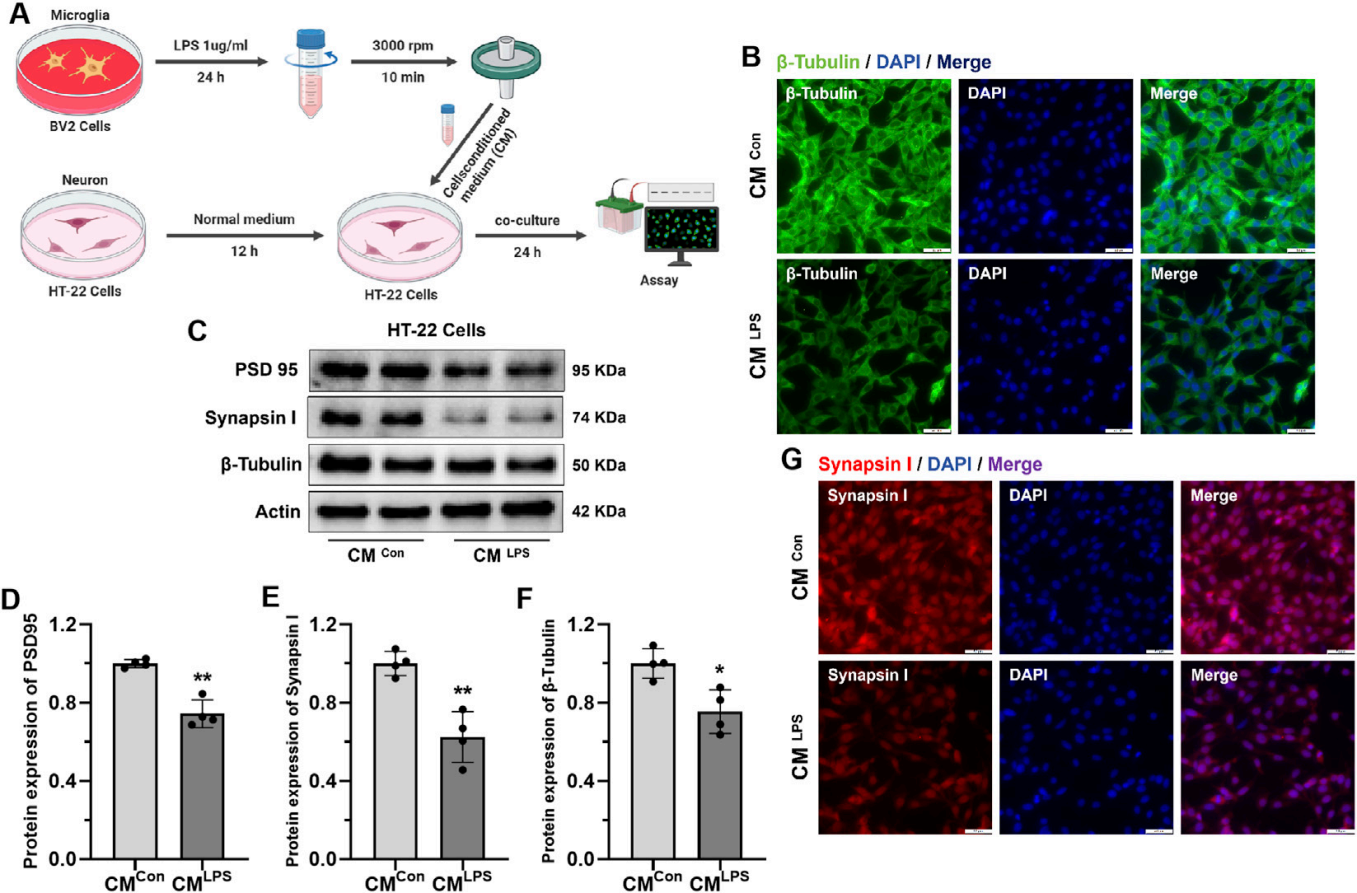
CM derived from LPS-challenged microglial cells induced neuron damage and synaptic plasticity impairment in HT-22 Cells. The data are presented as the mean ± SEM, and all the experiments were repeated with two separate passages of cells at least 4 times (n = 4). ^*^ *P* < 0.05 and ^**^ *P* < 0.01 compared with the CM^Con^ group. **(A)** Outline of the experimental design for CM-treated HT-22 cells. **(B)** Images of immunofluorescence staining for β-Tubulin (green) in CM-treated HT-22 cells. **(C–F)** Protein expression of synaptic plasticity related proteins PSD95, Synapsin I and β-Tubulin in CM-treated HT-22 cells. **(G)** Images of immunofluorescence staining for Synapsin I (red) in CM-treated HT-22 cells.

Next, we evaluated whether the conditioned medium (CM) derived from LPS-induced microglial cells could induce neuronal damage and synaptic plasticity impairment in neuron cells. For this purpose, HT-22 cells were treated with 50% CM derived from LPS-stimulated BV-2 cells for 24 h, and the neuronal damage was then observed (Figure 3A). Immunofluorescence analysis showed that HT-22 cells treated with BV2-CM^LPS^ exhibited cellular deformation, and markedly weaker β-Tubulin fluorescence intensity than cells treated with control medium (CM^Con^) (Figure 3B).

Moreover, the protein expression levels of synaptic plasticity related proteins PSD95 (*P* < 0.01) and Synapsin I (*P* < 0.01), as well as β-Tubulin protein (*P* < 0.05), were significantly decreased in BV2-CM^LPS^ treated HT-22 cells compared with CM^Con^ treated cells (Figures 3C–F). Consistently, decreased protein expression levels of PSD95 (*P* < 0.01) and Synapsin I (*P* < 0.01) were also found in SH-SY5Y cells treated with HMC3-CM^LPS^, as shown in Supplementary Figure S3. Furthermore, immunofluorescence staining showed that the positive fluorescence intensity of Synapsin I was also decreased in BV2-CM^LPS^ treated HT-22 cells when compared with CM^Con^ treated cells (Figure 3G). These findings suggest that CM derived from LPS-stimulated BV2 cells could induce microglia-mediated neuronal damage and synaptic plasticity impairment in HT-22 cells.

### 3.4 Caly showed potential target engagement with TLR4 protein

**FIGURE 4:**
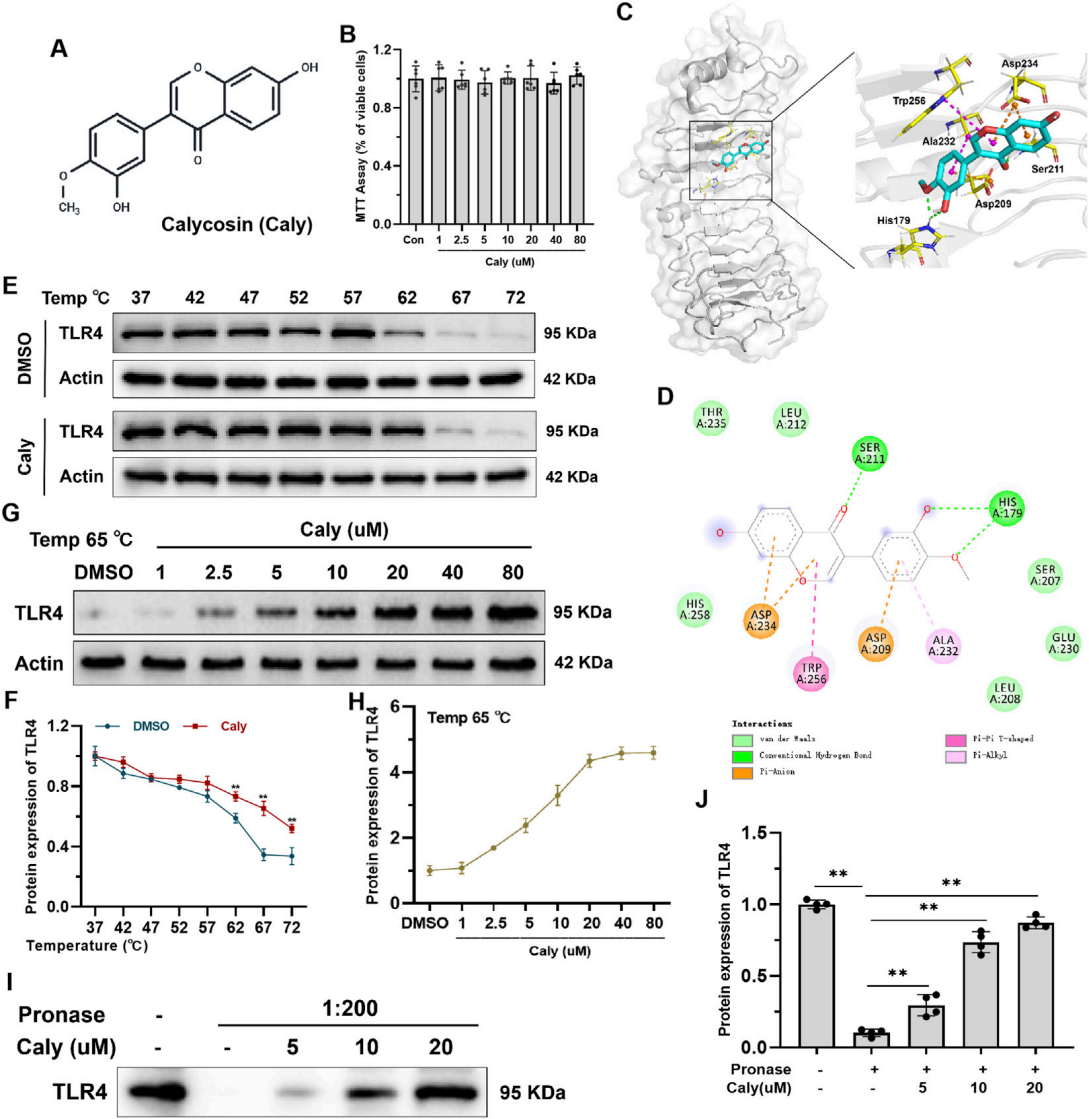
Caly showed potential target engagement with TLR4 protein. The data are presented as the mean ± SEM, and all the experiments were repeated with two separate passages of cells at least 4 times (n = 4). ^*^ *P* < 0.05 and ^**^ *P* < 0.01 compared with the DMSO group. **(A)** Chemical structure of Caly (20575-57-9; molecular weight, 284.26; purity, 99.91%). **(B)** Dose effect of Caly (1, 2.5, 5, 10, 20, 40 or 80 uM) on the proliferation of BV2 cells. **(C,D)** The 3D and 2D image revealed that the docking pocket and its internal binding bonds between Caly and TLR4. **(E,F)** BV2 cells treated with Caly (10 μM) and exposed to temperatures from 37 °C to 72 °C to assess TLR4 protein stability. **(G,H)** BV2 cells tincubated with different concentrations of Caly (1–80 μM) at 65 °C to assess TLR4 protein stability. **(I,J)** BV2 cells tincubated with Caly (5, 10, or 20 μM) and subjected to Pronase 1:200 to assess TLR4 protein stability.

It has been reported that Caly (Figure 4A) could exhibit various pharmacological activities including anti-inflammatory and neuroprotective effects, and the results of MTT assay also showed that there was no significant changes in the survival rate of BV2 cells when treatment with Caly at concentrations ranging from 1 to 80 μM for 24 h, as shown in Figure 4B.

The molecular docking analysis was performed to elucidate the binding mode of Caly with TLR4. As shown in Figures 4C,D, Caly is anchored in the TLR4 binding pocket primarily through a robust hydrogen bond network. The imidazole side chain of His179 acts as a dual donor, engaging its HE2 proton with O18 (2.06 Å) and O20 (2.20 Å) of the ligand, while Ser211 donates an additional hydrogen bond to O11 (2.53 Å). These three conventional hydrogen bonds precisely position the isoflavone core for complementary electrostatic and hydrophobic contacts. Beyond hydrogen bonding, multiple anion–π interactions significantly stabilize the complex. The negatively charged carboxylate groups of Asp209 and Asp234 approach the electron-rich aromatic ring of calycosin at distances of 3.55 Å and 3.36 Å, respectively, creating strong electrostatic attractions that enhance binding affinity. Hydrophobic packing further reinforces the interface: Trp256 engages in a T-shaped π–π stacking interaction (centroid distance 4.82 Å), and Ala232 forms a π-alkyl contact (4.16 Å). Collectively, the multivalent interaction pattern-a tight hydrogen-bonding network, dual aspartate-mediated anion-π stabilization, and hydrophobic packing, which suggests that the isoflavone core is optimally substituted with hydrogen bond acceptors at positions O18 and O20. Additionally, the calculated binding energy for Caly and TLR4 was −6.46 kcal/mol, which indicating a moderate-to-strong interaction.

To further validate the Caly and TLR4 interactions in a native cellular context, the CETSA and DARTS analyses were performed. Results of CETSA experiments showed the apparent aggregation temperatures with either Caly or DMSO (Figure 4E), and the substantial shifts demonstrated the binding of Caly and TLR4. As shown in Figure 4F, the protein damage in the DMSO group increased as the temperature increased. After Caly bound to TLT4, the thermal stabilization of TLR4 increased, especially at 62 °C, and this change was more significant than that in the DMSO group (*P* < 0.01). Moreover, as shown in Figures 4G,H, the results of ITDRF_CETSA_ assay showed a concentration-dependent increase in the thermal stabilization of TLR4 following Caly treatment, supporting dose-dependent target engagement between Caly and TLR4 in a cellular context. Similarly, in the DARTS assay, Caly treatment could reduce the protease sensitivity of TLR4 after pronase E digestion, as indicated by the increased retention of TLR4 protein compared with the control group. The protective effect against proteolytic degradation was significant (*P* < 0.01) (Figures 4I,J), further supporting the target engagement between Caly and TLR4 protein.

### 3.5 Caly ameliorated LPS-induced cytotoxicity and inflammatory responses in BV2 microglial cells

**FIGURE 5:**
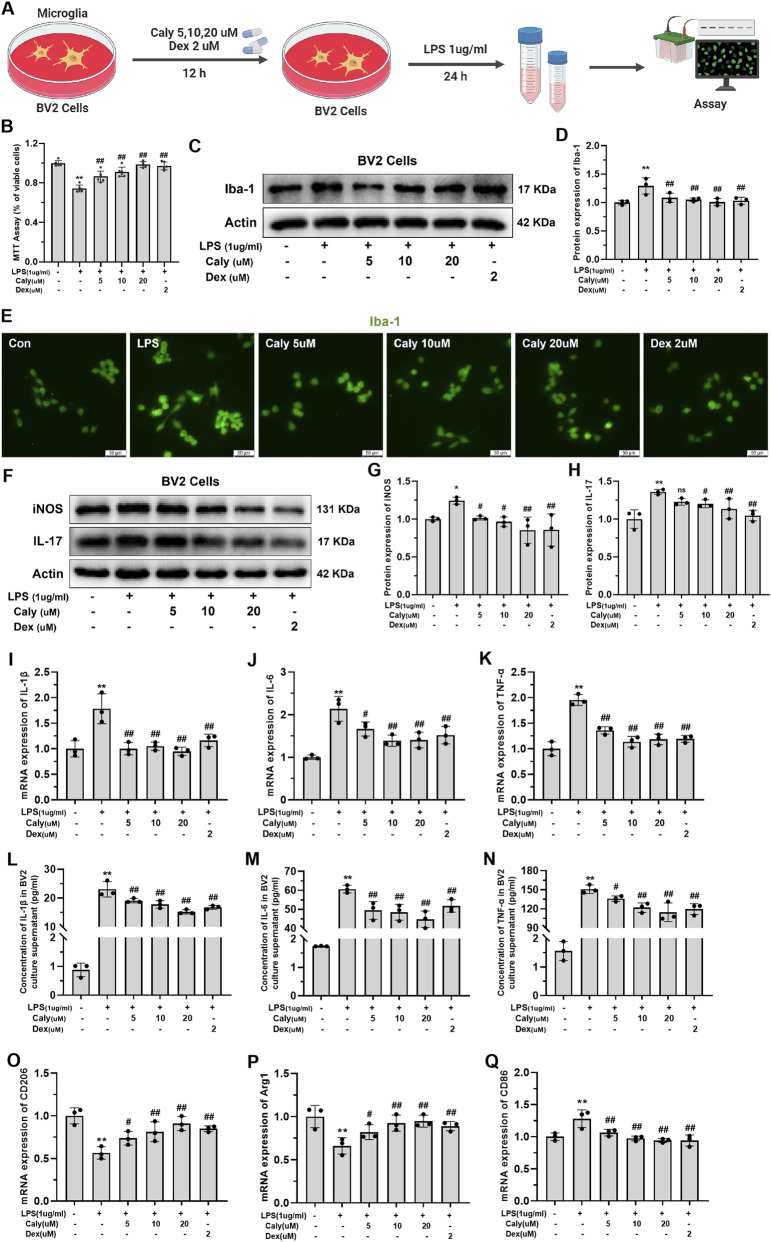
Caly ameliorated LPS-induced cytotoxicity and Inflammatory response in BV2 microglial cells. The data are presented as the mean ± SEM, and all the experiments were repeated with two separate passages of cells at least 3 times (n = 3). ^*^ *P* < 0.05 and ^**^ *P* < 0.01 compared with the Con group. ^#^ *P* < 0.05 and ^##^ *P* < 0.01 compared with the LPS group. **(A)** Outline of the experimental design for BV2 cells and Caly treatment. **(B)** Effect of treatment with Caly (5, 10, 20 μM) and Dex (2 μM) on the proliferation of LPS-induced BV2 cells. **(C,D)** Protein expression of Iba-1 in LPS-induced and Caly-treated BV2 cells. **(E)** Images of immunofluorescence staining for Iba-1 (green) in LPS-induced and Caly-treated BV2 cells. **(F–H)** Protein expression of iNOS and IL-17 in LPS-induced and Caly-treated BV2 cells. **(I–K)** mRNA expression levels of IL-1β, IL-6 and TNF-α in LPS-induced and Caly-treated BV2 cells. **(L–N)** Concentrations of IL-1β, IL-6 and TNF-α in the culture supernatant of BV2 cells after LPS stimulation and Caly treatment. **(O–Q)** mRNA expression levels of CD206, CD86 and Arg1 in LPS-induced and Caly-treated BV2 cells.

To investigate whether Caly exerted protective effects against LPS-induced cytotoxicity and inflammatory responses, the Caly (5, 10, 20 μM) were given to LPS-challenged BV2 cells (Figure 5A). As shown in Figure 5B, the cell viability of BV2 cells was reduced to approximately 70% after LPS stimulation compared with the Con group (P < 0.01), whereas it was significantly increased in the Caly (5, 10, 20 μM) or Dex (2 μM) treated groups as compared with LPS-induced cells (P < 0.01). As shown in Figures 5C,D, the protein expression level of Iba-1 was decreased in cells treatment with Caly (5, 10, 20 μM) or Dex (2 μM) compared with LPS-induced cells (P < 0.01). Moreover, immunofluorescence analysis showed that treatment with Caly (5, 10, 20 μM) or Dex (2 μM) alleviated cell deformation and reduced Iba-1 fluorescence intensity in LPS-activated BV2 cells (Figure 5E).

Further investigation demonstrated that treatment with Caly (5, 10, 20 μM) or Dex (2 μM) reversed the LPS-induced increases in the proteins expression levels of the pro-inflammatory mediator iNOS (*P* < 0.05 or *P* < 0.01) (Figure 5G) and the inflammation-associated mediator IL-17 (*P* < 0.05 or *P* < 0.01) (Figure 5H) in BV2 cells, as shown in Figures 5F–H. Consistent with this, treatment with Caly could also reduce the increased protein expression levels of iNOS (*P* < 0.01) and IL-17 (*P* < 0.05 or *P* < 0.01) in LPS-challenged HMC3 cells, as shown in Supplementary Figure S4.

Furthermore, quantitative real-time PCR analysis showed that treatment with Caly (5, 10, 20 μM) or Dex (2 μM) markedly decreased the LPS-induced mRNA expression levels of IL-1β (*P* < 0.01), IL-6 (*P* < 0.05 or *P* < 0.01) and TNF-α (*P* < 0.01) in BV2 cells, as shown in Figures 5I–K. Consistent with these, results of ELISA further confirmed that treatment with Caly significantly reduced the elevated concentrations of IL-1β (*P* < 0.01), IL-6 (*P* < 0.01), and TNF-α (*P* < 0.01) in the culture supernatants of LPS-stimulated BV2 cells (Figures 5L–N), indicating that Caly inhibited not only the gene expression but also the secretion of pro-inflammatory cytokines. Similarly, Caly treatment also suppressed the LPS-induced upregulation of IL-1β (*P* < 0.01), IL-6 (*P* < 0.01) and TNF-α (*P* < 0.01) mRNA expression levels in HMC3 cells, as shown in Supplementary Figure S5.

In addition, Caly (5, 10, 20 μM) or Dex (2 μM) treatment increased the mRNA expression levels of the anti-inflammatory markers phenotype-associated CD206 (*P* < 0.05 or *P* < 0.01) and Arg1 (*P* < 0.05 or *P* < 0.01), while reducing the expression of the pro-inflammatory marker CD86 (*P* < 0.01) in LPS-stimulated BV2 cells, as shown in Figures 5O–Q. Taken together, these findings suggest that Caly could ameliorate LPS-induced cytotoxicity and inflammatory responses in BV2 microglial cells.

### 3.6 Caly normalized the LPS-challenged imbalanced expression of TLR4/MyD88/NF-κB signaling pathway in BV2 microglial cells

**FIGURE 6:**
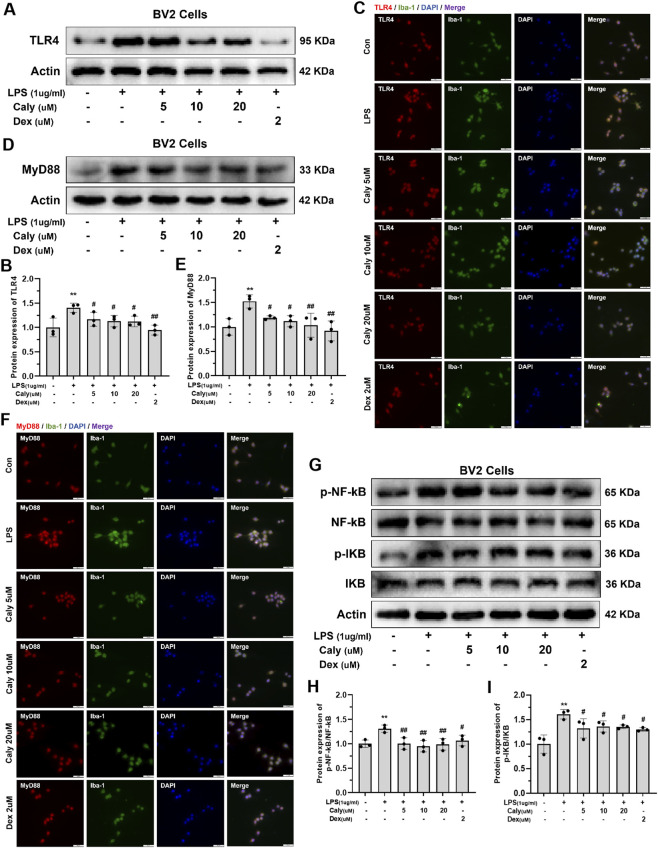
Caly normalized LPS-challenged imbalanced expression of TLR4/MyD88/NF-κB signaling pathway in BV2 microglial cells. The data are presented as the mean ± SEM, and all the experiments were repeated with two separate passages of cells at least 3 times (n=3). ^*^ *P* < 0.05 and ^**^ *P* < 0.01 compared with the Con group. ^#^ *P* < 0.05 and ^##^ *P* < 0.01 compared with the LPS group. **(A,B)** Protein expression of TLR4 in LPS-induced and Caly-treated BV2 cells. **(C)** Images of immunofluorescence double staining for TLR4 (red) and Iba-1 (green) iin LPS-induced and Caly-treated BV2 cells. **(D,E)** Protein expression of MyD88 in LPS-induced and Caly-treated BV2 cells. **(F)** Images of immunofluorescence double staining for MyD88 (red) and Iba-1 (green) iin LPS-induced and Caly-treated BV2 cells. **(G–I)** Protein expression of p-NF-kB/NF-kB and p-IKB/IKB in LPS-induced and Caly-treated BV2 cells.

As shown in Figures 6A,B, the protein expression level of TLR4 was markedly decreased in cells treatment with Caly (5, 10, 20 μM) or Dex (2 μM) compared with LPS-induced cells (*P* < 0.05 or *P* < 0.01). In addition, double immunofluorescence staining was performed to evaluate the co-localization of TLR4 with the activated microglial marker Iba-1. The results showed that the positive fluorescence intensity of TLR4/Iba-1 was stronger in LPS-induced BV2 cells than that in the Con group, whereas treatment with Caly (5, 10, 20 μM) or Dex (2 μM) reduced the fluorescence intensity of TLR4/Iba-1 (Figure 6C). Consistent with this, treatment with Caly (5, 10, 20 μM) or Dex (2 μM) also reversed the increased protein expression level of MyD88 (*P* < 0.05 or *P* < 0.01) (Figures 6D,E) and as well as the enhanced positive fluorescence intensity of MyD88/Iba-1 in LPS-induced BV2 cells (Figure 6F). Similarly, decreased protein expression levels of TLR4 (*P* < 0.01) and MyD88 (*P* < 0.01) were also found in Caly-treated HMC3 cells when compared with LPS-induced cells, as shown in Supplementary Figure S6.

Moreover, as shown in Figures 6G–I, the increased relative protein expression levels of p-NF-κB/NF-κB (*P* < 0.05 or *P* < 0.01) (Figure 6H) and p-IKB/IKB (*P* < 0.05) (Figure 6I) were both reversed after treatment with Caly (5, 10, 20 μM) or Dex (2 μM) in LPS-stimulated BV2 cells. Taken together, these results suggested that Caly could modulate the LPS-challenged imbalanced expression of TLR4/MyD88/NF-κB signaling pathway in BV2 microglial cells.

### 3.7 CM derived from Caly-treated cells protected against microglia-mediated neurotoxicity and synaptic plasticity impairment in HT-22 cells

**FIGURE 7:**
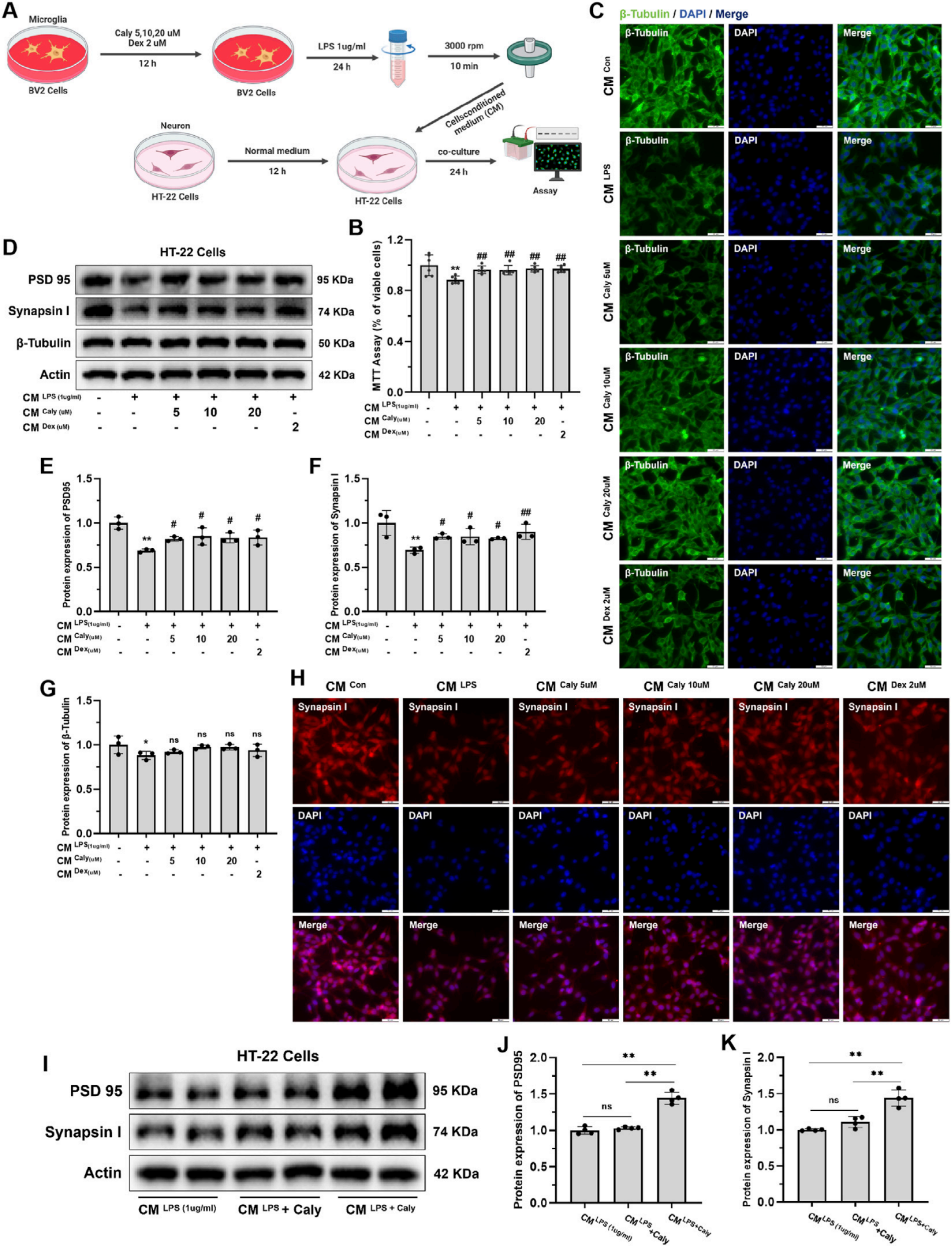
CM derived from Caly-treated cells protected against microglia-mediated neurotoxicity and synaptic plasticity impairment in HT-22 cells. The data are presented as the mean ± SEM, and all the experiments were repeated with two separate passages of cells at least 3 times (n = 3). ^*^ *P* < 0.05 and ^**^ *P* < 0.01 compared with the CM^Con^ group. ^#^ *P* < 0.05 and ^##^ *P* < 0.01 compared with the CM^LPS^ group. **(A)** Outline of the experimental design for CM-treated HT-22 cells. **(B)** Effect of treatment with CM derived from Caly-treated BV2 cells on the proliferation of HT-22 cells. **(C)** Images of immunofluorescence staining for β-Tubulin (green) in CM-treated HT-22 cells. **(D–G)** Protein expression of synaptic plasticity related proteins PSD95, Synapsin I and β-Tubulin in CM-treated HT-22 cells. **(H)** Images of immunofluorescence staining for Synapsin I (red) in CM-treated HT-22 cells. **(I–K)** Protein expression of synaptic plasticity related proteins PSD95 and Synapsin I in HT-22 cells treated with CM or Caly.

Since Caly attenuated LPS-induced inflammatory responses in microglia, we next determined whether Caly treatment could protect against neuronal toxicity induced by LPS-activated microglia (Figure 7A). The MTT assay showed that HT-22 cells treated with CM derived from LPS-induced BV2 cells exhibited a significant decrease in cell viability when compared with CM^Con^ treated cells (*P* < 0.05) (Figure 7B). In contrast, HT-22 cells treated with CM collected from BV2 cells incubated with Caly (5, 10, 20 μM) showed an increased cell viability compared with the CM^LPS^ treated group (*P* < 0.05) (Figure 7B). Immunofluorescence analysis also showed that BV2-CM collected from the Caly (5, 10, 20 μM)-treated cells increased the fluorescence intensity of β-Tubulin in HT-22 cells when compared to the BV2-CM^LPS^ treated group (Figure 7C). These results suggest that Caly treatment of BV2 cells may attenuate the indirect cytotoxicity induced by LPS-activated microglia in HT-22 cells.

Further studies revealed that although there was no significant difference in the β-Tubulin protein expressions between the BV2-CM^LPS^ treated group and the BV2-CM^Caly^ (5, 10, 20 μM) treated groups (Figure 7G), the protein expression levels of synaptic plasticity related proteins PSD95 (*P* < 0.05) (Figure 7E) and Synapsin I (*P* < 0.05 or *P* < 0.01) (Figure 7F) were remarkably increased in HT-22 cells treated with CM from BV2 cells incubated with Caly (5, 10, 20 μM) or Dex (2 μM) when compared with the CM^LPS^ treated cells, as shown in Figures 7D–G. Consistent with this, increased protein expression levels of PSD95 (*P* < 0.01) and Synapsin I (*P* < 0.01) were also found in SH-SY5Y cells treated with CM from HMC3 cells that been incubated with Caly (5, 10, 20 μM) when compared with that in the CM^LPS^ treated group, as shown in Supplementary Figure S7. Furthermore, in the immunofluorescence staining assay, the positive fluorescence intensity of Synapsin I were also increased in HT-22 cells after treated with CM incubated with Caly (5, 10, 20 μM) or Dex (2 μM) when compared with cells treated with CM from LPS-induced BV2 cells (Figure 7H).

In addition, to exclude the potential interference of residual Caly carried over in the CM, we also constructed a matched control group, in which HT-22 cells were treated with CM collected from LPS-stimulated BV2 cells without Caly intervention, and fresh Caly at 10 μM was added directly to this CM immediately before application to HT-22 cells. As shown in Figures 7I–K, direct addition of Caly to CM derived from LPS-induced BV2 cells did not exert a significant neuroprotective effect on damaged HT-22 cells, as evidenced by the lack of significant restoration of PSD95 and Synapsin I expression. These results suggested that the protective effects of CM from Caly-treated BV2 cells were not mainly due to residual Caly directly acting on HT-22 cells, but were more likely attributable to the suppression of microglia-derived neurotoxic inflammatory mediators by Caly.

## 4 Discussion

**FIGURE 8:**
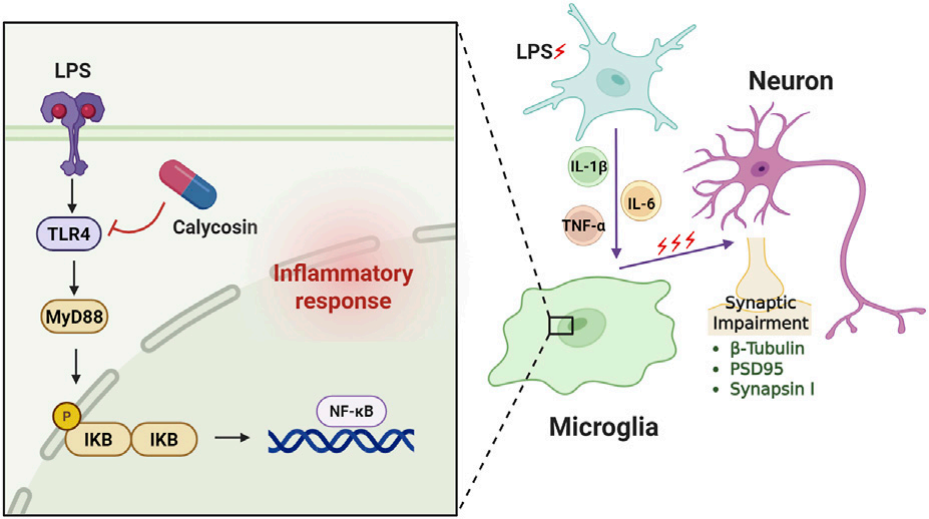
Effect of Caly against LPS-induced inflammatory response in BV2 cells and microglia-mediated synaptic impairment in HT-22 cells. Stimulation with LPS could induce excessive activation of BV2 cells, and co-culture with conditioned medium (CM) derived from LPS-stimulated BV-2 cells could cause cellular damage and synaptic plasticity imbalance in HT-22 cells. Treatment with Caly could alleviate the cytotoxicity and inflammatory response, reverse the imbalanced expression of TLR4/MyD88/NF-κB signaling pathway induced by LPS in BV2 cells, and improve synaptic plasticity impairment in microglia-mediated HT-22 cells.

Many studies have shown that the inflammatory responses play an important role in the pathophysiology of various neurodegenerative diseases (Kwon and Koh, 2020), and activation of the TLR4/MyD88/NF-κB signaling pathway has been implicated in the neuroinflammatory processes (Wei et al., 2024; Jiang et al., 2025). In the present study, our findings suggest that Caly attenuated the production and secretion of pro-inflammatory mediators and downregulated the TLR4/MyD88/NF-κB signaling pathway in LPS-stimulated BV2 cells. In addition, CM derived from Caly-treated BV2 cells partially alleviated microglia-mediated neuronal injury and synaptic impairment in HT-22 cells (Figure 8). These results indicate that Caly may exert protective effects against LPS-induced microglial inflammatory activation and microglia-mediated neuronal damage, at least partly through modulation of the TLR4/MyD88/NF-κB signaling pathway.

Neuroinflammation is a defensive response in the CNS, representing the body’s attempt to reduce injury and promote repair (Castellani et al., 2023). However, dysregulation of the neuroinflammatory process may lead to the propagation of neuropathology and has been documented in the progression of numerous neurological disorders (Amanollahi et al., 2023; Shi and Yong, 2025). Microglia, as the resident innate immune cells in the CNS, play a central role in mediating the neuroinflammatory responses in neurological disorders (Leng and Edison, 2021). Activated microglia could secrete a repertoire of pro-inflammatory mediators, amplify the neuroinflammatory responses and contribute to the disruption of CNS homeostasis (AmeliMojarad and AmeliMojarad, 2024). As one of the most commonly used inflammatory stimuli in biological research, lipopolysaccharide (LPS) has been widely used to induce systemic or local inflammatory responses. Stimulation by LPS has been reported to elicit microglia activation and neuronal damage (Kim et al., 2024; Xu D. et al., 2023). In the present study, our results showed that LPS could induce a declined cell viability in a dose-dependent manner, accompanied by the obvious morphological changes and significant inflammatory response in BV2 cells, as indicated by the increased mRNA expression levels of IL-1β, IL-6 and TNF-α. In addition to these intracellular inflammatory changes, ELISA results further showed that the concentrations of secreted IL-1β, IL-6, and TNF-α were significantly increased in the culture supernatant of LPS-stimulated BV2 cells, confirming that LPS promoted the release of pro-inflammatory cytokines into the conditioned medium. Moreover, LPS stimulation decreased the expression levels of the anti-inflammatory phenotype-associated markers CD206 and Arg1, while increasing the expression of the pro-inflammatory phenotype-associated markers CD86 and iNOS, indicating that LPS not only induced inflammatory mediator production and secretion, but also shifted BV2 cells toward a pro-inflammatory phenotype. In line with the reported effect of LPS on cell damage and neuroinflammation (Choi et al., 2024), these results indicated that LPS (1 μg/mL) could effectively simulate the pro-inflammatory state of microglia in BV2 cells, which provides a reliable *in vitro* tool for subsequent studies on neuroinflammation and drug intervention.

Moreover, protein expression of IL-17 was also included and detected based on our previous RNA-seq screening results in the present study, and the result showed an increased protein levels of IL-17 in LPS-induced BV2 cells, which suggested the involvement of IL-17-related inflammatory changes in the LPS-induced microglial inflammatory response. Although IL-17 is mainly recognized as a T-cell-associated cytokine, accumulating evidence suggests that IL-17/IL-17A may participate in neuroinflammatory processes and interact with microglial inflammatory responses under specific pathological conditions. Previous studies have reported that microglia could produce IL-17 in response to specific inflammatory stimulation and that IL-17 could enhance microglial production of inflammatory mediators such as IL-6 and nitric oxide (Kawanokuchi et al., 2008). Besides, in LPS-induced neuroinflammation models, IL-17A has also been implicated in microglial activation, pro-inflammatory cytokine production, cognitive impairment, and synaptic marker reduction (Sun et al., 2015). Other studies have also indicated that IL-17 could act on CNS-resident glial cells and amplify neuroinflammatory responses (Chen et al., 2020; Jurga et al., 2020).

The relationship between microglial inflammatory activation and neuronal synaptic impairment is increasingly recognized as an important component of neuroinflammation-related CNS dysfunction (Frosch et al., 2025; Marinelli et al., 2019). It has been demonstrated that activated microglia could influence neurons not only through the release of soluble inflammatory mediators, but also through bidirectional microglia-neuron communication (Frosch et al., 2025; Qian et al., 2024; Shan et al., 2025). In the present study, we established an indirect co-culture system in which HT-22 neuronal cells were treated with conditioned medium (CM) collected from LPS-stimulated BV2 cells. The results showed that CM derived from LPS-activated microglia significantly impaired neuronal cytoskeletal integrity and synaptic protein expression in HT-22 cells, suggesting that pro-inflammatory mediators secreted by activated microglia exert neurotoxic effects on neurons. As a core component of the neuronal microtubule cytoskeleton, β-Tubulin plays an essential role in maintaining neuronal morphology and structural stability (Pero et al., 2023). It has been reported that β-Tubulin levels are significantly decreased in proximity to amyloid plaques in AD patients, which may compromise synaptic integrity (Fracassi et al., 2023). Moreover, synaptic proteins are critical for synaptic plasticity and neuronal function, among which PSD95 and Synapsin I play key roles in synaptic formation and function (Qi et al., 2021). Decreased expression of PSD95 and Synapsin I has been observed in the hippocampus of LPS-induced depression-like mice and PA-induced HT-22 cells in our previous studies, and these changes were closely associated with neuronal damage and cognitive impairment (Gao et al., 2023; Fang et al., 2019; Xu D. et al., 2023). Consistent with these previous findings, the present study also showed decreased expression of β-Tubulin, PSD95 and Synapsin I in BV2-CM^LPS^ treated HT-22 cells, further supporting that activated microglia-mediated neuroinflammation could disrupt the basic structural scaffold of neurons and impair synaptic protein expression.

As a classic synthetic glucocorticoid, dexamethasone (Dex) has long been used clinically for the treatment of acute bacterial meningitis and other infectious neurological disorders (Swildens et al., 2022). In addition to suppressing excessive neuroinflammatory responses, Dex has been reported to exert immunosuppressive and neuroprotective effects in various CNS diseases (Sun et al., 2023; Wei et al., 2022; Jeong et al., 2021). Moreover, high doses of Dex has been shown to achieve detectable concentrations in the CNS (Yu et al., 2017). Previous studies have demonstrated that Dex could modulate microglia activation by inhibiting the NF-κB signaling pathway, reducing the secretion of pro-inflammatory cytokines, and promoting a shift from a pro-inflammatory phenotype toward an anti-inflammatory phenotype. It was worth noting that the immunomodulatory effects of Dex appear to be dose-dependent. For example, prolonged exposure to high-dose Dex has been reported to suppress M2 microglial polarization and reduce dendritic spine loss through the GR/JAK1/STAT3 signaling pathway (Yang et al., 2025). In addition, Dex has been shown to markedly inhibite LPS-induced BV2 microglial activation through regulation of the TRAF6/TAK-1/JNK signaling pathways (Hui et al., 2020). In the present study, Dex was selected as the positive control. Consistent with previous reports, treatment with Dex (2 μM) effectively attenuated LPS-induced microglial hyperactivation, suppressed the release of neurotoxic inflammatory mediators, and alleviated microglia-mediated neurotoxicity in neuronal cells.

In recent years, bioactive compounds derived from traditional Chinese medicines have gained increasing attention in neuroinflammation research due to the multitarget regulatory effects and favorable safety profiles (Wang et al., 2024; Li et al., 2024; Wang et al., 2022). Calycosin (Caly) is a major active isoflavonoid derived from Astragali aadix and has been widely reported to exert neuroprotective effects in various CNS disorder models, including cerebral ischemia/reperfusion injury (CIRI) (Xu S. et al., 2023), Alzheimer’s disease (AD) (An et al., 2022), and Parkinson’s disease (PD) (Pan et al., 2021). It has been reported that Caly could ameliorate neurological deficits and reduce brain infarct volume in middle cerebral artery occlusion (MCAO) rats by inhibiting neuroinflammation (Yang et al., 2024). Another study demonstrated that Caly treatment attenuated neurological injury in post-stroke rats by modulating brain-derived neurotrophic factor (BDNF)-tyrosine kinase receptor B (TrkB) signaling pathways and microglial activation (Hsu et al., 2020). Moreover, Caly has been reported to reduce the tendency of microglia toward pro-inflammatory activation and inhibit inflammatory responses by regulating the HMGB1/TLR4/NF-κB signaling pathway (Li et al., 2023). In the present study, we investigated whether Caly could ameliorate LPS-induced neuroinflammation in BV2 cells and attenuate microglia-mediated synaptic impairment in HT-22 cells. Consistent with the regulatory effects of Dex, treatment with Caly (5, 10, 20 μM) attenuated LPS-induced microglial hyperactivation, neuroinflammation and microglia-mediated neurotoxicity, as indicated by the downregulation of IL-17, increased of anti-inflammatory phenotype-associated markers CD206 and Arg1, decreased of pro-inflammatory marker CD86 and iNOS, as well as the reduced mRNA expression and concentrations of secreted IL-1β, IL-6, and TNF-α in LPS-stimulated BV2 cells. These findings suggest that Caly may shift LPS-activated microglia toward a less pro-inflammatory phenotype.

Furthermore, CM derived from Caly-treated BV2 cells partially restored the expression of β-Tubulin, PSD95, and Synapsin I in HT-22 cells exposed to inflammatory microglial CM. Excessive production of IL-1β, IL-6, TNF-α, nitric oxide, and ROS by activated microglia may disrupt neuronal cytoskeletal stability, impair synaptic protein expression, and disturb synaptic transmission (Ferro et al., 2021; Cornell et al., 2022). In particular, recent studies have shown that microglia-derived cytokines could modulate neuronal excitability and synaptic plasticity in an activity-dependent manner. For example, microglial cytokines have been reported to participate in synaptic plasticity induced by repetitive magnetic stimulation (Eichler et al., 2023), while TNF-α-mediated synaptic modulation is also influenced by microglial state (Kleidonas et al., 2023). In addition, direct microglia-neuron interactions, including membrane contact-dependent communication, CX3CL1/CX3CR1 signaling, complement-related synaptic remodeling, and microglial surveillance of synaptic compartments, are considered essential mechanisms for maintaining neuronal homeostasis and synaptic integrity (Frosch et al., 2025; Kang et al., 2025). Therefore, the restoration of β-tubulin, PSD95 and Synapsin I expression observed in HT-22 cells treated with CM incubated with Caly may be associated with the reduction of microglia-derived inflammatory and neurotoxic mediators. Taken together, these results suggest that Caly attenuates LPS-induced microglial neuroinflammation and microglia-mediated neuronal injury in in vitro model, which provides experimental evidence for the potential pharmacological relevance of Caly in neuroinflammation-associated CNS disorders.

At the molecular level, toll-like receptor 4 (TLR4) is an important transmembrane receptor involved in inflammatory processes, which mainly expresses in microglia and astrocytes and has been implicated in various CNS diseases (Wang et al., 2023). TLR4 has been reported to activate nuclear factor-kappa B (NF-κB) through the myeloid differentiation factor 88 (MyD88)-dependent pathway, ultimately inducing the production of inflammatory cytokines (Lwin et al., 2021). Increasing evidence has that regulation of the TLR4/MyD88/NF-κB pathway in microglia is closely associated with neuroinflammation and cognitive impairment (Yu et al., 2023; Wei et al., 2024). Consistent with previous studies (Hu et al., 2020; Feng et al., 2025), the present study also found that LPS stimulation could activate the TLR4/MyD88/NF-κB signaling pathway in BV2 cells. It has been reported that Caly could reduce oxidative stress markers and downregulate pro-inflammatory cytokines through the HMGB1/TLR4/NF-κB pathway in CIRI (Li et al., 2023; Yang et al., 2024). Moreover, in PD mice, Caly has been shown to prevent dopaminergic neuronal death and reduce neuroinflammation by inhibiting the TLR4/NF-κB and MAPK pathways (Yang et al., 2019). Besides, the network pharmacology analysis in our previous study suggested that Caly may regulate inflammation in pulmonary fibrosis through the TLR4/MyD88/NF-κB signaling pathway. Consistently, in the present study, Caly treatment significantly decreased the protein expression level of TLR4 and MyD88, and reduced the phosphorylation levels of NF-κB and IKB in LPS-stimulated BV2 cells. Furthermore, molecular docking, CETSA, ITDRF_CETSA_ and DARTS assays collectively supported potential target engagement between Caly and TLR4 protein, which provides a possible molecular basis for the regulation of the TLR4/MyD88/NF-κB signaling pathway by Caly. Collectively, these results suggest that Caly may inhibit LPS-induced microglial activation and neuroinflammatory responses, at least partly by regulating the TLR4/MyD88/NF-κB signaling pathway.

There were several limitations in the present study. Firstly, the protective effect of Caly was entirely observed in immortalized murine cell lines (BV2 microglia and HT-22 neuronal cell lines). We fully recognize that immortalized cell lines exhibit inherent differences from primary cells in terms of gene expression profiles, inflammatory response characteristics, and synaptic formation capacity. In particular, BV2 cells may respond differently in some inflammatory pathways compared to primary microglia, and HT-22 cells may lack the complex dendritic morphology and functional synaptic networks characteristic of mature hippocampal neurons. Although we have partially strengthened the generalizability of our conclusions through cross-species validation using human cell lines HMC3 (human microglia cells) and SH-SY5Y (human neuronal cells), more kinds of cell models, especially primary mouse microglia and hippocampal neurons should be used to verify the direct effect of Caly on microglia-synapse crosstalk, and animal models of neuroinflammation (such as LPS-induced mice or rats models) would be better. Secondly, the mechanistic relationship between reduced microglial inflammatory activation and neuronal synaptic modulation was mainly inferred from an indirect CM-based cell model. Although CM derived from LPS-stimulated BV2 cells induced neuronal damage and decreased PSD95 and Synapsin I expression in HT-22 cells, it cannot directly demonstrate physical microglia-neuron interactions, real-time synaptic transmission, or neuronal network activity. Further studies using primary microglia-neuron co-cultures, organotypic hippocampal slice cultures, electrophysiological recordings, confocal or live-cell imaging of microglia-neuron contacts are required to clarify the caUnited Statesl relationship between microglial inflammatory suppression and neuronal synaptic functional recovery. Finally, although the target engagement between Caly and TLR4 protein, as well as the imbalanced expression levels of TLR4/MyD88/NF-κB signaling pathway have been detected in the present study, the possible caUnited Statesl relationship and precise mechanism beyond these changes have not been uncovered, more detailed investigations should be conducted in the future study.

## 5 Conclusion

In conclusion, the present study demonstrated that Caly could exert potential effects on suppressing LPS-induced microglia activation and inflammatory responses in immortalized BV2 cells by regulating TLR4/MyD88/NF-κB signaling pathway, and attenuating microglia-mediated neuronal injury and synaptic impairment in immortalized HT-22 cells (Figure 8). The additional validation in HMC3 and SH-SY5Y cells further supports the reproducibility of these effects across murine and human cell-line systems. These findings provide *in vitro* evidence that Caly may modulate microglia-mediated neuroinflammatory responses and neuronal damage, and suggest its potential pharmacological relevance for neuroinflammation-associated neurodegenerative disorders. However, because the current findings were obtained from immortalized *in vitro* models, further studies using primary microglia, primary neurons, organotypic hippocampal slice cultures, and *in vivo* animal models are needed to confirm whether Caly exerts similar anti-neuroinflammatory and synaptic protective effects under more physiologically relevant conditions.

## Data Availability

The raw data supporting the conclusions of this article will be made available by the authors, without undue reservation.
