# Supplementary material for: Calycosin attenuates LPS-induced microglia inflammatory responses and microglia-mediated synaptic impairment via modulation of TLR4/MyD88/NF-κB signaling pathway
Source: Front Pharmacol. 2026 Jul 28;17:1817366. doi: 10.3389/fphar.2026.1817366 (PMC13457696; doi:10.3389/fphar.2026.1817366)
Supplement: Supplementary file 1 [file DataSheet1.pdf]

Calycosin attenuates LPS-induced microglia inflammatory responses and microglia-mediated synaptic impairment via modulation of TLR4/MyD88/NF-κB signaling pathway

Xinran Gao[1,2], Ruiyang Wang[1,2], Hejuntao Chen[1,2], Xu Chen[1,2], Xiaojuan Wang[1,2*]

[1] The First Affiliated Hospital of Anhui University of Science and Technology, Huainan, 232001, PR China

[2] The First Clinical Medical School, Anhui University of Science and Technology, Huainan, 232001, PR China

*** Corresponding author:**

Xiaojuan Wang, Prof.

The First Affiliated Hospital of Anhui University of Science and Technology, 203 Huaibin Road, Huainan, 232001, PR China

Telephone number: 0086 0554 3320237

E-mail: wxjaust@163.com

Methods

Cell culture

Human microglial cell line HMC3 and human neuroblastoma cell line SH-SY5Y were obtained from Wuhan Shangen Biotechnology Co., Ltd., China. Cells were maintained in the HMC3 cell-specific complete medium (DMEM, GZ11006, Servicebio) and SH-SY5Y cell-specific complete medium (DMEM, GZ11004, Servicebio) respectively, and incubated at 37 °C in a 5% carbon dioxide incubator.

Cell activation and treatment

Exponentially growing HMC3 cells (5×10^5 cells/well) were seeded in 6-well plates and cultured until they reached approximately 60% confluence. The cells were then treated with LPS at a concentration of 1 μg/mL for 24 h. To evaluate the treatment effects of Caly, HMC3 cells were pretreated with Caly (5, 10, or 20 μM) or Dex (2 μM) for 12 h, followed by exposure to LPS (1 μg/mL) for an additional 24 h.

Conditioned medium collection and treatment of SH-SY5Y cells

To prepare HMC3 conditioned medium (CM), HMC3 cells were treated with Caly (5, 10, 20 μM) and stimulated with LPS (1 μg/mL). The culture supernatants were then collected and filtered through a 0.22 μm filter to remove cells and cell debris. SH-SY5Y cells were seeded in 6-well plates at a density of 5×10^5 cells/well and incubated for 12 h. Subsequently, 50% of the culture medium in each well was removed and replaced with an equal volume of HMC3-CM for 24 h.

Supply Figure 1

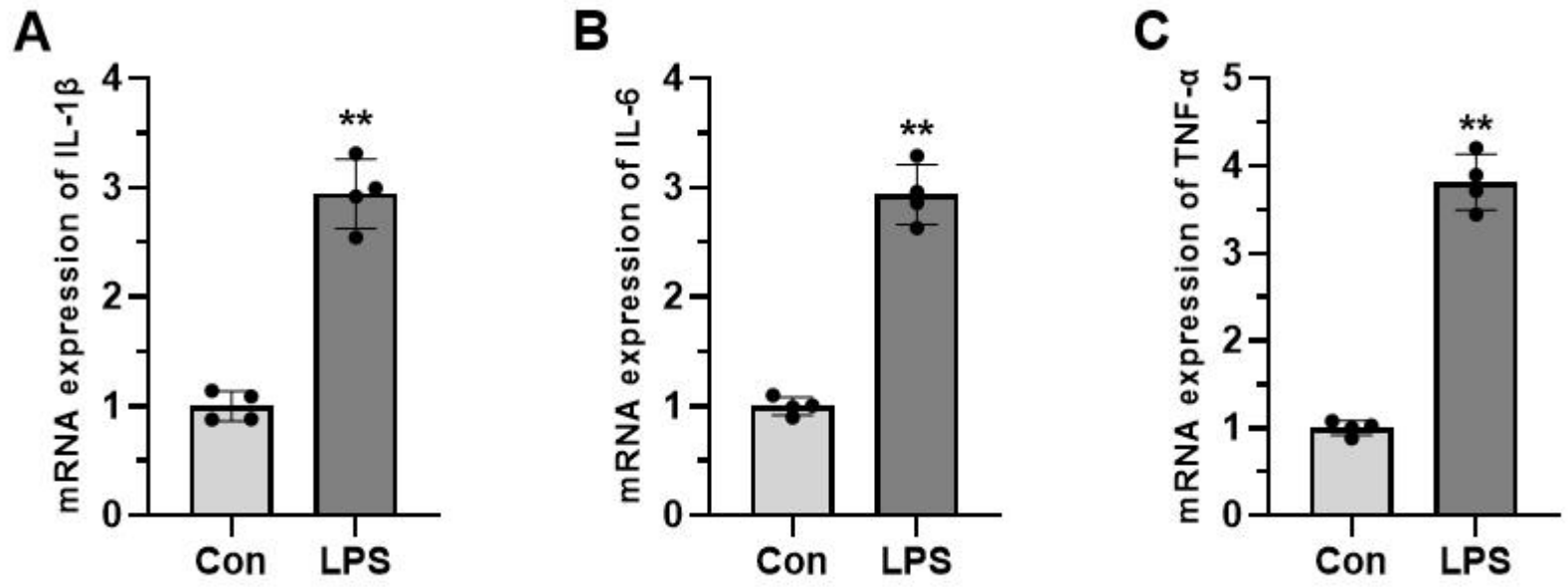

Figure S1 Increased mRNA expression levels of IL-1β, IL-6 and TNF-α in LPS-induced HMC3 cells.

A mRNA expression levels of IL-1β. **B** mRNA expression levels of IL-6. **C** mRNA expression levels of TNF-α.

The data are presented as the mean ± SEM, and all the experiments were repeated with two separate passages of cells at least 4 times (n=4). $^{**}P < 0.01$ compared with the Con group.

Supply Figure 2

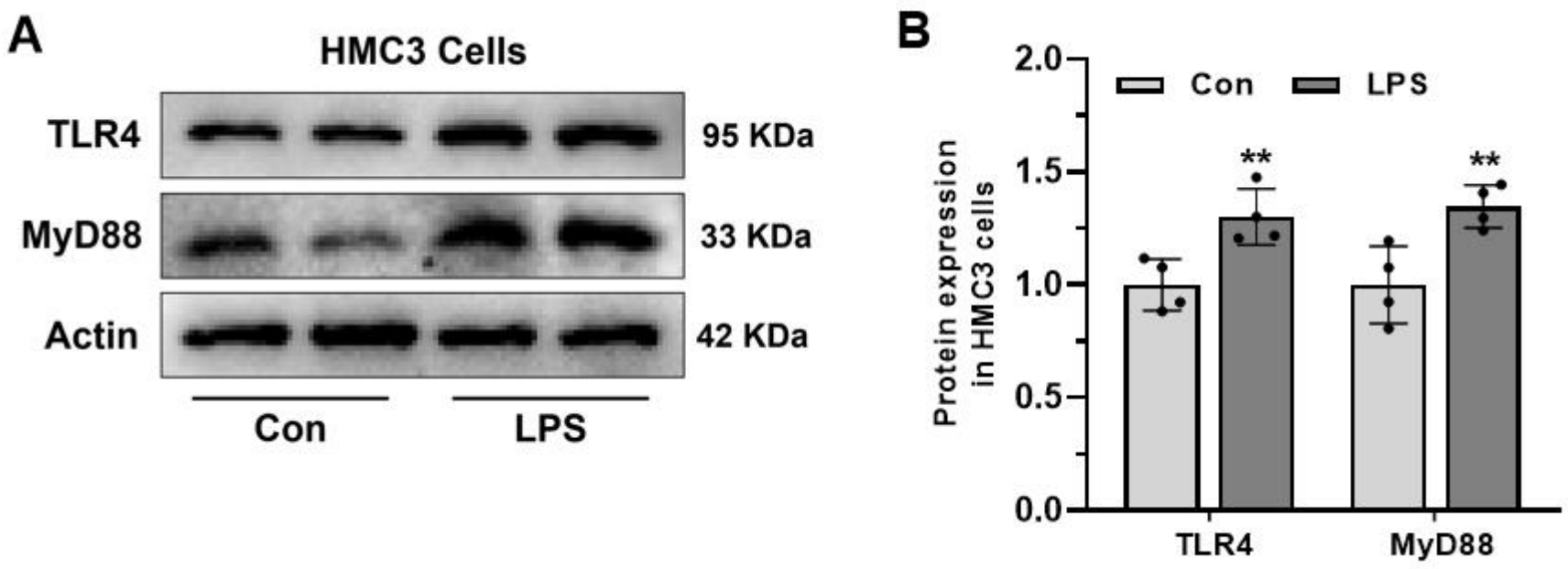

Figure S2 Increased protein expression levels of TLR4 and MyD88 in LPS-induced HMC3 cells.

A Representative western blotting bands. **B** Relative protein levels of TLR4 and MyD88 in HMC3 cells.

The data are presented as the mean ± SEM, and all the experiments were repeated with two separate passages of cells at least 4 times (n=4). $^{**}P < 0.01$ compared with the Con group.

Supply Figure 3

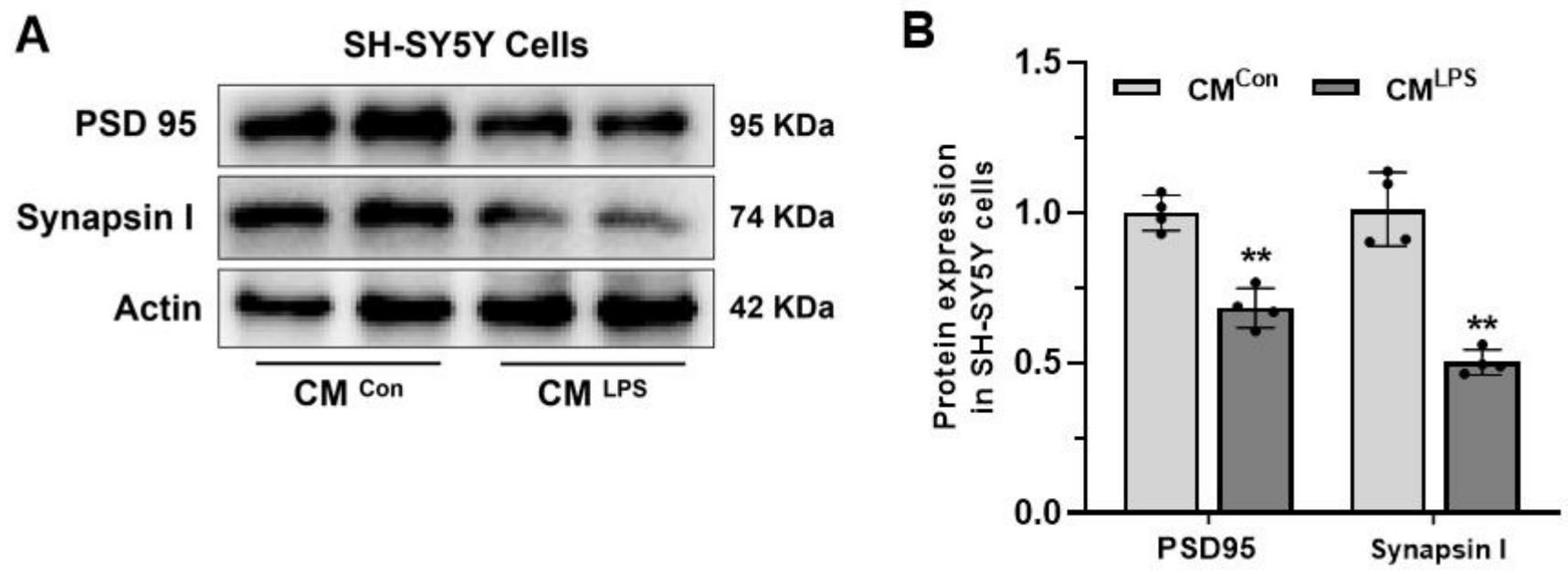

Figure S3 Decreased protein expression levels of PSD95 and Synapsin I in HMC3-CM^{LPS} treated SH-SY5Y cells.

A Representative western blotting bands. **B** Relative protein levels of PSD95 and Synapsin I in SH-SY5Y cells.

The data are presented as the mean ± SEM, and all the experiments were repeated with two separate passages of cells at least 4 times (n=4). $^{**}P < 0.01$ compared with the CM^{Con} group.

Supply Figure 4

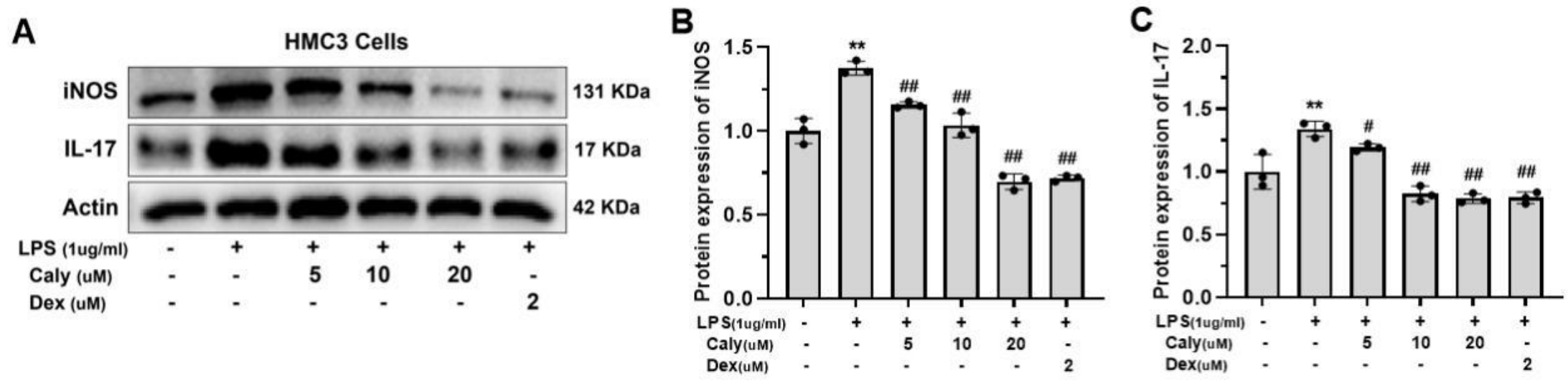

Figure S4 Caly normalized the increased protein expression levels of iNOS and IL-17 in LPS-challenged HMC3 cells.

A Representative western blotting bands. **B-C** Relative protein levels of iNOS and IL-17 in HMC3 cells.

The data are presented as the mean ± SEM, and all the experiments were repeated with two separate passages of cells at least 3 times (n=3). $^{**}P < 0.01$ compared with the Con group. $^{\#}P < 0.05$ and $^{\#\#}P < 0.01$ compared with the LPS group.

Supply Figure 5

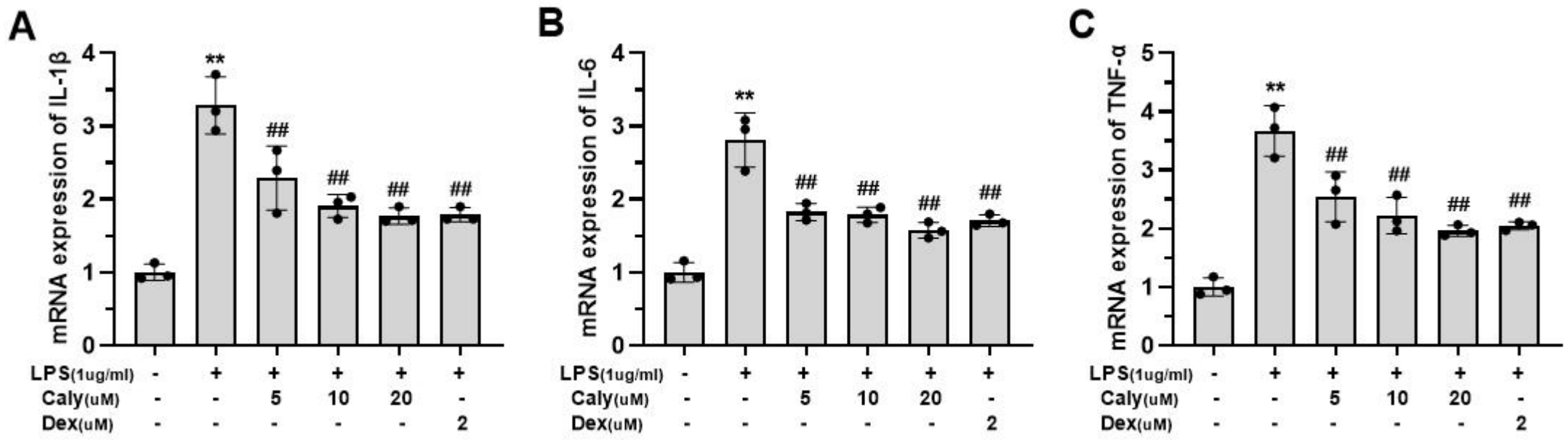

Figure S5 Caly normalized the increased mRNA expression levels of IL-1β, IL-6 and TNF-α in LPS-challenged HMC3 cells.

A mRNA expression levels of IL-1β. **B** mRNA expression levels of IL-6. **C** mRNA expression levels of TNF-α.

The data are presented as the mean ± SEM, and all the experiments were repeated with two separate passages of cells at least 3 times (n=3). $^{**}P < 0.01$ compared with the Con group. $^{##}P < 0.01$ compared with the LPS group.

Supply Figure 6

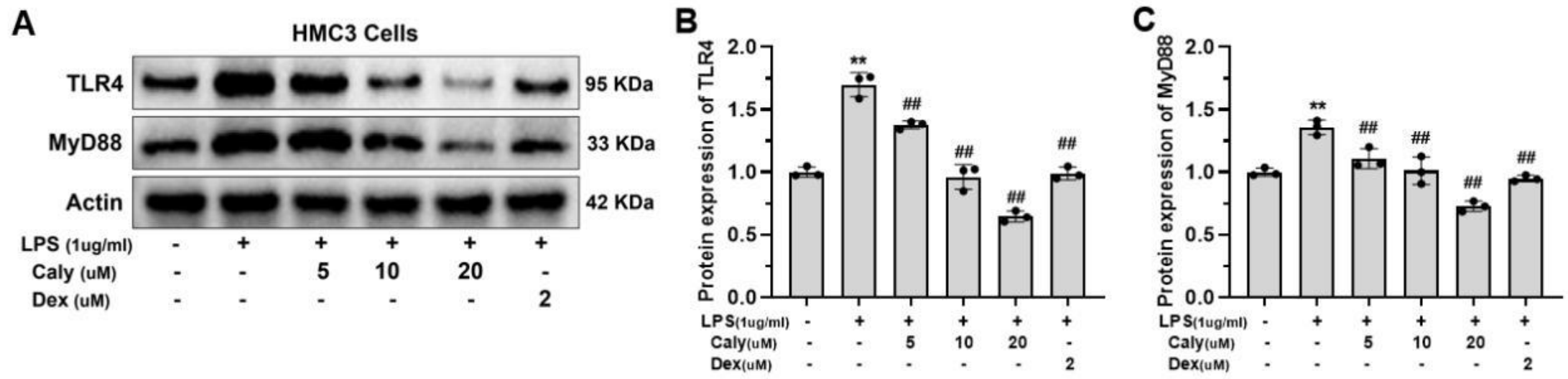

Figure S6 Caly normalized the increased protein expression levels of TLR4 and MyD88 in LPS-challenged HMC3 cells.

A Representative western blotting bands. **B-C** Relative protein levels of TLR4 and MyD88 in HMC3 cells.

The data are presented as the mean ± SEM, and all the experiments were repeated with two separate passages of cells at least 3 times (n=3). $^{**}P < 0.01$ compared with the Con group. $^{\#\#}P < 0.01$ compared with the LPS group.

Supply Figure 7

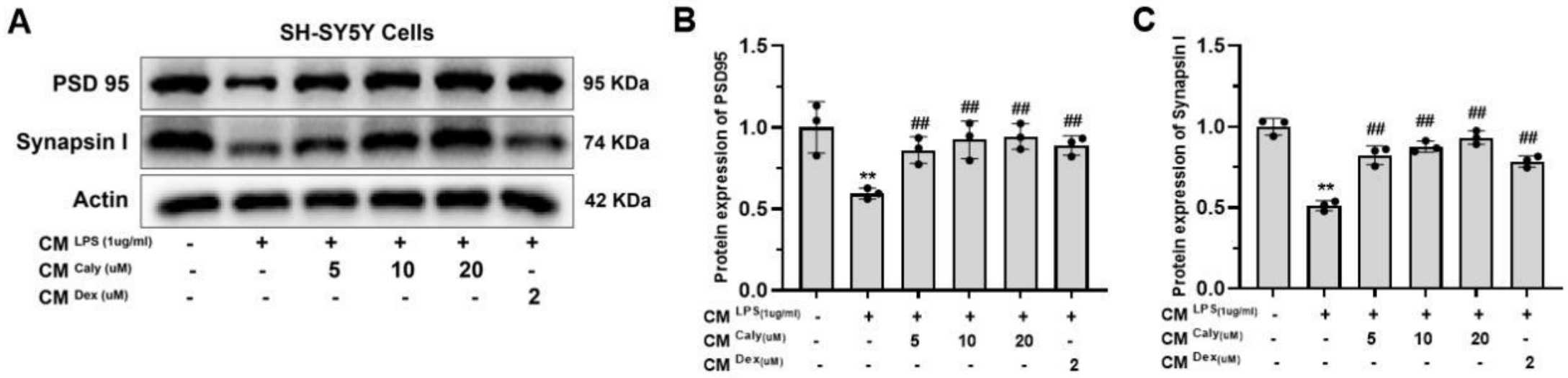

Figure S7 CM derived from Caly-treated cells normalized the decreased protein expression levels of PSD95 and Synapsin I in HMC3-CM^{LPS} treated SH-SY5Y cells. **A** Representative western blotting bands. **B-C** Relative protein levels of PSD95 and Synapsin I in SH-SY5Y cells.

The data are presented as the mean ± SEM, and all the experiments were repeated with two separate passages of cells at least 3 times (n=3). $^{**}P < 0.01$ compared with the CM^{Con} group. $^{\#\#}P < 0.01$ compared with the CM^{LPS} group.
