# Supplementary material for: Calycosin attenuates LPS-induced microglia inflammatory responses and microglia-mediated synaptic impairment via modulation of TLR4/MyD88/NF-κB signaling pathway
Source: Front Pharmacol. 2026 Jul 28;17:1817366. doi: 10.3389/fphar.2026.1817366 (PMC13457696; doi:10.3389/fphar.2026.1817366)
Supplement: Supplementary file 2 [file Table1.docx]

Table 1 The primer sequences

| Source | Gene | Forward primer | Reverse primer |
| --- | --- | --- | --- |
| mouse | IL-1β | GCTTCAGGCAGGCAGTAT | ACAAACCGCTTTTCCATCT |
|  | IL-6 | GAGGATACCACTCCCAACAGACC | AAGTGCATCATCGTTGTTCATACA |
|  | TNF-α | CATCTTCTCAAAATTCGAGTGACAA | TGGGAGTAGACAAGGTACAACCC |
|  | CD206 | TGTACGCAGTGGTTGGCAGTG | GCTCTGATGATGGACTTCCTGGTAG |
|  | CD86 | CAGCACGGACTTGAACAACC | CTCCACGGAAACAGCATCTGA |
|  | Arg1 | CAGTCCCTGGCTTATGGTTACCC | GAAGACAGCAGAGGAGGTGAAGAG |
|  | β-actin | AGTGTGACGTTGACATCCGT | TGCTAGGAGCCAGAGCAGTA |
